# Pathogenic Fungi Regulate Immunity by Inducing Neutrophilic Myeloid-Derived Suppressor Cells

**DOI:** 10.1016/j.chom.2015.02.007

**Published:** 2015-04-08

**Authors:** Nikolaus Rieber, Anurag Singh, Hasan Öz, Melanie Carevic, Maria Bouzani, Jorge Amich, Michael Ost, Zhiyong Ye, Marlene Ballbach, Iris Schäfer, Markus Mezger, Sascha N. Klimosch, Alexander N.R. Weber, Rupert Handgretinger, Sven Krappmann, Johannes Liese, Maik Engeholm, Rebecca Schüle, Helmut Rainer Salih, Laszlo Marodi, Carsten Speckmann, Bodo Grimbacher, Jürgen Ruland, Gordon D. Brown, Andreas Beilhack, Juergen Loeffler, Dominik Hartl

**Affiliations:** 1 Department of Pediatrics I, University of Tübingen, 72076 Tübingen, Germany; 2 Department of Medicine II, University of Würzburg, 97080 Würzburg, Germany; 3 IZKF Research Group for Experimental Stem Cell Transplantation, Department of Medicine II, 97080 Würzburg, Germany; 4 Department of Pediatrics, Yong Loo Lin School of Medicine, National University of Singapore, Singapore 119077, Singapore; 5 Institute of Cell Biology, Department of Immunology, University of Tübingen, 72076 Tübingen, Germany; 6 Microbiology Institute – Clinical Microbiology, Immunology and Hygiene, University Hospital of Erlangen and Friedrich-Alexander University Erlangen-Nürnberg, 91054 Erlangen, Germany; 7 Department of Pediatrics, University of Würzburg, 97080 Würzburg, Germany; 8 Department of Neurology, University of Tübingen, 72076 Tübingen, Germany; 9 Department of Oncology, University of Tübingen, 72076 Tübingen, Germany; 10 Department of Infectious and Pediatric Immunology, Medical and Health Science Center, University of Debrecen, 4032 Debrecen, Hungary; 11 Centre of Chronic Immunodeficiency (CCI), University Medical Center Freiburg and University of Freiburg, 79106 Freiburg, Germany; 12 Institut für Klinische Chemie und Pathobiochemie, Klinikum rechts der Isar, Technische Universität München, 81675 Munich, Germany; 13 Aberdeen Fungal Group, Section of Immunology and Infection, University of Aberdeen, AB24 3FX Aberdeen, UK

## Abstract

Despite continuous contact with fungi, immunocompetent individuals rarely develop pro-inflammatory antifungal immune responses. The underlying tolerogenic mechanisms are incompletely understood. Using both mouse models and human patients, we show that infection with the human pathogenic fungi *Aspergillus fumigatus* and *Candida albicans* induces a distinct subset of neutrophilic myeloid-derived suppressor cells (MDSCs), which functionally suppress T and NK cell responses. Mechanistically, pathogenic fungi induce neutrophilic MDSCs through the pattern recognition receptor Dectin-1 and its downstream adaptor protein CARD9. Fungal MDSC induction is further dependent on pathways downstream of Dectin-1 signaling, notably reactive oxygen species (ROS) generation as well as caspase-8 activity and interleukin-1 (IL-1) production. Additionally, exogenous IL-1β induces MDSCs to comparable levels observed during *C. albicans* infection. Adoptive transfer and survival experiments show that MDSCs are protective during invasive *C. albicans* infection, but not *A. fumigatus* infection. These studies define an innate immune mechanism by which pathogenic fungi regulate host defense.

## Highlights

• Pathogenic fungi induce myeloid-derived suppressor cells (MDSCs)
• MDSC induction involves Dectin-1/CARD9, ROS, caspase-8, and IL-1
• MDSCs dampen T and NK cell immune responses
• Adoptive transfer of MDSCs improves survival in *Candida* infection in vivo

## Graphical Abstract

**Figure:**
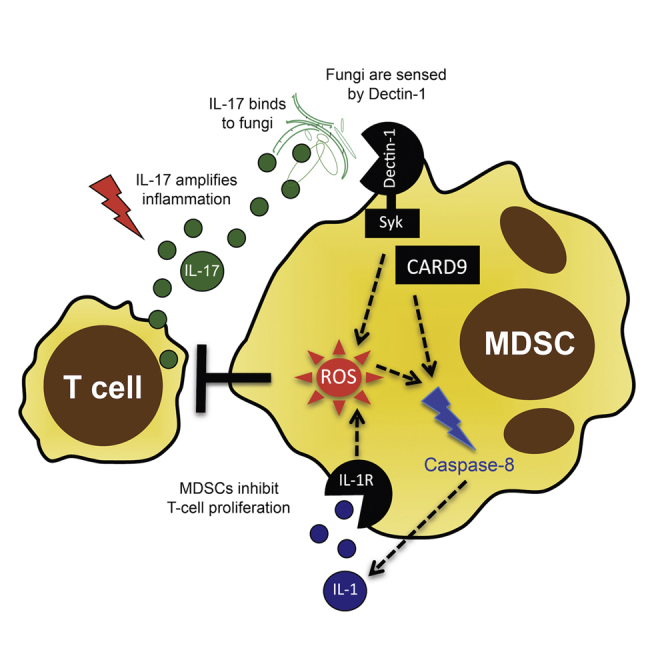

## Teaser

Myeloid-derived suppressor cells (MDSCs) are innate immune cells that suppress T cell responses. Rieber et al. show that pathogenic fungi *Aspergillus fumigatus* and *Candida albicans* induce MDSCs through mechanisms involving Dectin-1/CARD as well as downstream ROS and IL-1β production, and that transfer of MDSCs protects against invasive *Candida* infection.

## Introduction

At mucosal sites, the human immune system is faced continuously with microbes, rendering fine-tuned immune responses essential to protect against pathogenic, while maintaining tolerance against harmless, species. This immune balance is of particular relevance for fungi, inhaled daily as spores or present in the gut microflora as commensal yeasts (Romani, 2011). While immunocompetent individuals do not develop invasive fungal infections, infections are a major problem in patients undergoing immunosuppression, for instance, at solid organ or hematopoietic stem cell transplantation (Garcia-Vidal et al., 2013).

Fungi are recognized through pattern recognition receptors, mainly C-type lectin receptors (with Dectin-1 as the prototypic one) (Steele et al., 2005), toll-like receptors (TLRs), and pentraxin 3 (PTX3) (Garlanda et al., 2002; Werner et al., 2009). A certain level of inflammation is essential to control fungal infections (Brown, 2010), but hyperinflammatory responses seem to cause more harm than good to the host. Particularly, Th17-driven hyperinflammatory responses have been shown to promote fungal growth (Zelante et al., 2012), to impair fungal clearance, and to drive tissue damage (Romani et al., 2008; Zelante et al., 2007). Generation of reactive oxygen species (ROS), indoleamine 2,3-dioxygenase (IDO) activity, and activation of the TIR domain-containing adaptor-inducing interferon-β (TRIF) pathway were found to limit hyperinflammatory responses toward *Aspergillus fumigatus* (Romani, 2011; Romani et al., 2009). Yet, the cellular mechanisms by which fungi control T cell activation and maintain tolerogenic host-pathogen bistability remain incompletely understood.

Myeloid-derived suppressor cells (MDSCs) are innate immune cells characterized by their capacity to suppress T cell responses (Gabrilovich and Nagaraj, 2009). MDSCs comprise a neutrophilic and a monocytic subset. While the functional impact of MDSCs in cancer is established, their role in host-pathogen interactions is poorly defined. We hypothesized that fungal infections induce MDSCs that modulate disease outcome.

## Results

**Figure 1:**
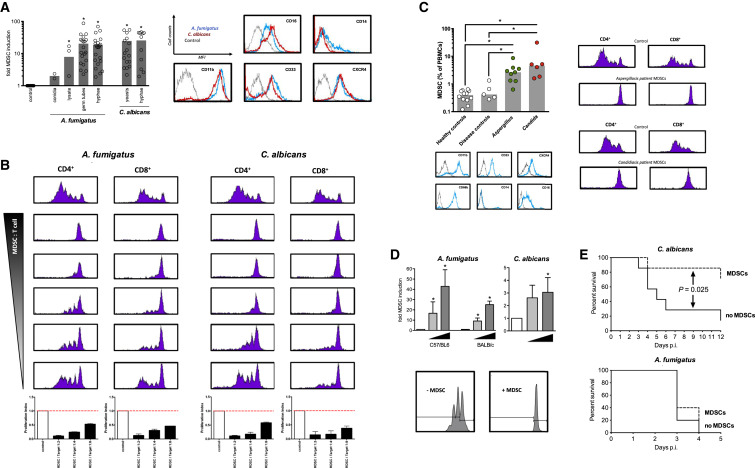
Fungi Induce Functional MDSCs In Vitro and In Vivo (A) Fungal morphotypes differentially induce MDSCs. Left panel: MDSCs were generated by incubating PBMCs (5 × 10^5^/ml) from healthy donors with medium only (negative control), or different morphotypes of *A. fumigatus* (conidia, 5 × 10^5^/ml; germ tubes, 1 × 10^5^/ml; hyphae, 1 × 10^5^/ml) or *C. albicans* (yeasts, 1 × 10^5^/ml; hyphae, 1 × 10^5^/ml). The x-fold induction of MDSCs compared to control conditions is depicted. ^∗^p < 0.05. Right panel: representative histograms of fungi-induced MDSCs (CD11b^+^CD33^+^CD14^−^CD16^+^CXCR4^+^). (B) Fungi-induced MDSCs suppress T cells. The suppressive effects of CD33^+^-MACS-isolated MDSCs were analyzed on CD4^+^ and CD8^+^ T cell proliferation. MDSCs were generated by incubating PBMCs (5 × 10^5^/ml) from healthy donors with *A. fumigatus* germ tubes (1 × 10^5^/ml) or *C. albicans* yeasts (1 × 10^5^/ml) for 6 days. Different MDSC-to-T cell ratios were assessed (1:2, 1:4, 1:6, 1:8, and 1:16). The lower bar graphs represent the proliferation index compared to control conditions as means ± SEM. (C) MDSCs in patients with fungal infections. Left panel: MDSCs were characterized as CD14^−^ cells expressing CD33, CD66b, CD16, CD11b, and CXCR4 in the PBMC fraction. The gray line shows unstained controls. MDSCs were quantified in peripheral blood from healthy controls, immunosuppressed patients without fungal infections (disease controls, n = 5), or immunosuppressed patients with invasive fungal infections (invasive *A. fumigatus* infections, n = 9, and invasive *C. albicans* infections, n = 6). ^∗^p < 0.05. Right panel: representative CFSE stainings, showing the effect of MDSCs isolated (MACS) from patients with invasive *A. fumigatus* infections (left) or invasive *C. albicans* infections (right) on CD4^+^ and CD8^+^ T cell proliferation. (D) Fungi induce MDSCs in mice in vivo. Upper left panel: C57/BL6 (n = 3 mice per treatment group) or BALB/c (n = 4 mice per treatment group) wild-type mice were not infected (white bars) or challenged intranasally with 1 × 10^4^ (light gray bar) or 1 × 10^6^ (dark gray bar) *A. fumigatus* conidia for 3 days. On the fourth day, a bronchoalveolar lavage (BAL) was performed, and CD11b^+^Ly6G^+^ MDSCs were quantified by FACS. The x-fold induction of CD11b^+^Ly6G^+^ MDSCs in the BAL compared to control non-infected conditions is depicted. ^∗^p < 0.05. Upper right panel: C57BL/6 mice were not infected (white bars) or injected via the lateral tail vein with 2.5 × 10^5^ (light gray bar) or 5 × 10^5^ (dark gray bar) blastospores of *C. albicans*. On the fifth day, mice were sacrificed, and CD11b^+^Ly6G^+^ MDSCs in the spleen were quantified by FACS. The x-fold induction of CD11b^+^Ly6G^+^ MDSCs in the spleen compared to control non-infected conditions is depicted. n = 5 mice per treatment group. ^∗^p < 0.05. Lower panel: bone marrow-isolated murine CD11b^+^Ly6G^+^ MDSCs were co-cultured for 3 days with T cells (CD4^+^ splenocytes) at a 1:2 (MDSCs:T cell) ratio. T cell proliferation was analyzed using the CFSE assay with and without MDSCs. (E) Adoptive transfer of MDSCs modulates survival in fungal infection. For adoptive transfer experiments, CD11b^+^Ly6G^+^ MDSCs were isolated from the bone marrow of BALB*/*c mice by MACS and checked for T cell suppression. In (A)–(D) bars represent means ± SEM. Upper panel: adoptive MDSC transfer was performed by intravenous (i.v.) injection of 5 × 10^6^ MDSCs per animal. Seven mice received MDSCs, while seven mice served as non-MDSC control animals. A total of 2 hr after the MDSC transfer, mice were i.v. injected with 1 × 10^5^ blastospores of *C. albicans*. Mice were weighed daily and monitored for survival and signs of morbidity. Lower panel: for invasive pulmonary *A. fumigatus* infection survival studies, mice were immunosuppressed by treatment with cyclophosphamide, and MDSC transfer was performed by i.v. injection of 4 × 10^6^ MDSCs per animal. Five mice received MDSCs, while five mice served as non-MDSC control animals. After the MDSC transfer, mice were challenged intranasally with 2 × 10^5^*A. fumigatus* conidia and were monitored for survival.

We analyzed the effect of the human-pathogenic fungi *A. fumigatus* and *C. albicans* on human immune cells and noticed the appearance of a cell population that was different from monocytes (CD14^−^), and expressed the myeloid markers CD33^+^, CD11b^+^, CD16^+^, and CXCR4 (Figures 1A and S1A). Fungi-induced myeloid cells strongly suppressed both CD4^+^ and CD8^+^ T cell proliferation in a dose-dependent manner (Figure 1B), which defines MDSCs. Fungi-induced MDSCs also suppressed innate natural killer (NK) cell responses, without affecting cell survival (Figure S2). In contrast to growth factor-induced MDSCs, fungi-induced MDSCs dampened Th2 responses, which play essential roles in fungal asthma (Kreindler et al., 2010) (Figure S1B). We quantified MDSCs in patients with invasive fungal infections and challenged mice with *A. fumigatus* or *C. albicans*. MDSCs accumulated in both *A. fumigatus*- and *C. albicans*-infected patients compared to healthy and disease control patients without fungal infections (Figure 1C). Murine studies further showed that systemic or pulmonary fungal challenge with *C. albicans* (invasive disseminated candidiasis) or *A. fumigatus* (pulmonary aspergillosis), as the clinically relevant routes of infection, dose-dependently triggered the recruitment of MDSCs in both immunocompetent and immunosuppressed conditions, with a stronger MDSC induction seen in immunocompetent animals (Figures 1D and S1C). MDSCs expressed neutrophilic markers in both man and mice, resembling the neutrophilic subtype of MDSCs (Rieber et al., 2013), while monocytic MDSC subsets were not induced (Figure S1D). Fungi-induced MDSCs functionally suppressed T cell proliferation (Figure 1C), while autologous conventional neutrophils failed to do (Figure S1E).

**Figure 2:**
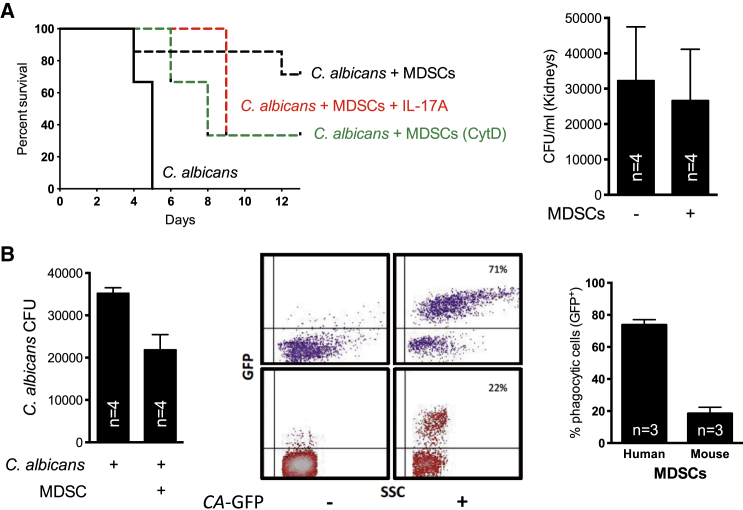
Antifungal Functions (A) In vivo. Left panel: survival in the invasive *C. albicans* infection model after adoptive MDSC transfer. Before adoptive transfer, isolated MDSCs were pretreated with cytochalasin D (CytD, 1 μg/ml, green line) or with recombinant mouse IL-17A protein (5 μg/mouse, red line). Right panel: *C. albicans* CFUs in kidneys of BALB/c mice 5 days after adoptive transfer of MDSCs. Bars represent means ± SD. (B) In vitro. Left panel: 1 × 10^6^ human MDSCs were co-cultured with 1 × 10^5^ serum opsonized *C. albicans* (10:1 ratio) for 3 hr at 37°C in RPMI. Serial dilutions were performed of the cell suspension, and 100 μl was plated onto YPD agar plates containing penicillin and streptomycin. Plates were incubated for 24–48 hr at 37°C, and CFU were enumerated. Middle and right panels: phagocytic capacity of human and murine MDSCs. Middle panel; upper (purple) FACS plots, isolated human granulocytic MDSCs (low-density CD66b^+^CD33^+^ cells) were co-cultured with or without GFP-labeled *C. albicans* (*CA*) spores (MOI = 1) in RPMI medium at 37°C for 90 min. Lower (red) FACS plots, isolated mouse granulocytic CD11b^+^Ly6G^+^ MDSCs were co-cultured with or without GFP-labeled *C. albicans* spores (MOI = 4) in RPMI medium at 37°C for 90 min. Representative dot blots are shown. Right panel: GFP expression/fluorescence of MDSCs was analyzed by FACS and is given in the right panel as percentage of GFP^+^ MDSCs.

We adoptively transferred T cell-suppressive neutrophilic MDSCs and monitored their impact on survival in fungal infection. While a single dose of adoptively transferred MDSCs was protective in systemic *C. albicans* infection, MDSCs had no impact on *A. fumigatus* infection (Figure 1E). Septic shock determines mortality in candidiasis (Spellberg et al., 2005), and the interplay of fungal growth and renal immunopathology was shown to correlate with host survival (Lionakis et al., 2011, 2013; Lionakis and Netea, 2013; Spellberg et al., 2003). Adoptively transferred MDSCs dampened renal T and NK cell activation and systemic Th17 and TNF-α cytokine responses (Figures S1F and S1G). Conversely, supplementing IL-17A dampened the MDSC-mediated protective effect (Figure 2A). Besides these immunomodulatory effects, MDSCs might also act directly antifungal, as our in vitro studies showed that they can phagocytose and kill fungi (Figure 2B). However, direct antifungal effects could hardly explain the beneficial effect of MDSCs in candidiasis: (i) adoptively transferred MDSCs had no effect on fungal burden in vivo (Figure 2A), (ii) inhibition of phagocytosis only partially diminished the protection conferred by MDSCs (Figure 2A), and (iii) MDSCs were exclusively protective in immunocompetent mice (*C. albicans* infection model), with no effect in immunosuppressed (neutropenic) mice (*A. fumigatus* infection model).

**Figure 3:**
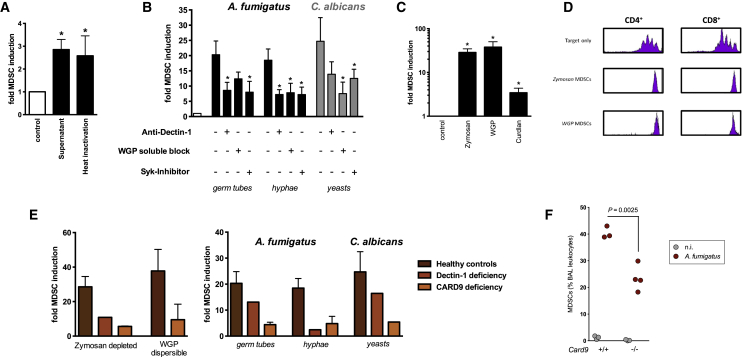
Fungi Induce MDSCs through a Dectin-1-, Syk-, and CARD9-Mediated Mechanism (A) Fungal factors mediating MDSC induction are heat resistant. MDSCs were generated by incubating PBMCs (5 × 10^5^/ml) from healthy donors with medium only (negative control), untreated, or heat-denatured (95°C, 30 min) supernatants (SNT) of *A. fumigatus* germ tubes (4%) for 6 days. The x-fold induction of MDSCs compared to control conditions is depicted. ^∗^p < 0.05 versus control conditions. (B) Dectin-1 and Syk are involved in fungal MDSC induction. MDSCs were generated in vitro by incubating isolated PBMCs (5 × 10^5^ cells/ml) with *A. fumigatus* germ tubes (1 × 10^5^/ml), hyphae (1 × 10^5^/ml), and *C. albicans* yeasts (1 × 10^5^/ml) for 6 days. Where indicated, PBMCs were pretreated for 60 min with anti-Dectin-1 blocking antibody (15 μg/ml), soluble WGP (1 mg/ml), and a Syk inhibitor (100 nM). ^∗^p < 0.05 blocking versus unblocked conditions. (C) Dectin-1/CARD9 ligands mimic fungal MDSC induction. MDSCs were generated in vitro by incubating isolated PBMCs with the Dectin-1/CARD9 ligands zymosan depleted (10 μg/ml), dispersible WGP (20 μg/ml), or curdlan (10 μg/ml). p < 0.05 versus control conditions. (D) Dectin-1/CARD9 ligands induce functional MDSCs. The suppressive effects of CD33^+^-MACS-isolated MDSCs were analyzed on CD4^+^ and CD8^+^ T cell proliferation (CFSE polyclonal proliferation assay). MDSCs were generated by incubating PBMCs (5 × 10^5^/ml) from healthy donors with zymosan depleted (10 μg/ml) or dispersible WGP (20 μg/ml). MDSC, T cell ratio was 1:6. (E) Fungal MDSC induction in patients with genetic Dectin-1 or CARD9 deficiency. Left panel: MDSCs were generated in vitro by incubating isolated PBMCs (5 × 10^5^ cells/ml) from healthy controls (n = 12), an individual with Dectin-1 deficiency, or patients with CARD9 deficiency (n = 2) with the Dectin-1/CARD9 ligands zymosan depleted (10 μg/ml) or dispersible WGP (20 μg/ml). Right panel: MDSCs were generated in vitro by incubating isolated PBMCs (5 × 10^5^ cells/ml) from healthy controls (n = 12), an individual with genetically proven Dectin-1 deficiency, or patients with CARD9 deficiency (n = 2) with different fungal morphotypes (1 × 10^5^ cells/ml) for 6 days. (F) CARD9 is involved in fungi-induced MDSC recruitment in vivo. *Card9*^−/−^ mice and age-matched wild-type mice were challenged intranasally with 1 × 10^6^*A. fumigatus* conidia for 3 days. On the fourth day, a BAL was performed, and CD11b^+^Ly6G^+^ MDSCs were quantified by flow cytometry. In (B), (C), and (E) bars represent means ± SEM.

The potency of *A. fumigatus* to induce MDSCs was most pronounced for germ tubes and hyphae, morphotypes characteristic for invasive fungal infections (Figure 1A) (Aimanianda et al., 2009; Hohl et al., 2005; Moyes et al., 2010). The MDSC-inducing fungal factor was present in conditioned supernatants and was heat resistant (Figure 3A), pointing to β-glucans as the bioactive component. We therefore focused on Dectin-1 as β-glucan receptor and key fungal sensing system in myeloid cells. Fungi-induced MDSCs expressed Dectin-1, and blocking Dectin-1 prior to fungal exposure diminished the MDSC-inducing effect, while blocking of TLR 4 had no effect (Figures 3B and S3). Furthermore, Dectin-1 receptor activation mimicked the generation of neutrophilic MDSCs phenotypically and functionally (Figures 3C and 3D). Dectin-1 receptor signaling was confirmed by blocking of the spleen tyrosine kinase Syk, which acts downstream of Dectin-1 (Figure 3B). We further used cells from human genetic Dectin-1 deficiency and used *Dectin-1* knockout mice for fungal infection models. The potential of fungi or fungal patterns to induce neutrophilic MDSCs was diminished in human and, albeit to a lesser extent, murine Dectin-1 deficiency (Figures 3E and S1D). We analyzed the role of caspase recruitment domain 9 (CARD9), a downstream adaptor protein and key transducer of Dectin-1 signaling, in fungi-mediated MDSC generation in patients with genetic *CARD9* deficiency and *Card9* knockout mice. These approaches demonstrated that CARD9 signaling was involved in fungal MDSC induction in the human and the murine system (Figures 3E and 3F).

**Figure 4:**
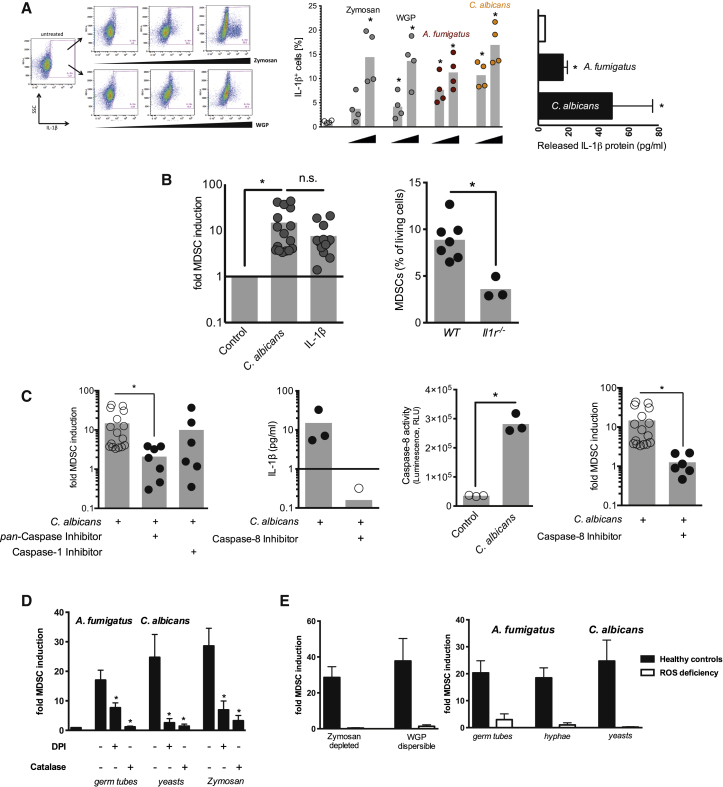
Fungal MDSC Induction Involves IL-1β, Caspase-8, and ROS (A) Intracellular accumulation and release of IL-1β. Left panel: gating strategy for intracellular cytokine staining. IL-1β was analyzed in CD33^+^ myeloid cells using intracellular cytokine staining and flow cytometry. Zymosan depleted (20, 100, and 500 μg/ml) and WGP dispersible (20, 100, and 500 μg/ml) were used for 1 hr to stimulate cytokine production. Middle panel: leukocytes isolated from healthy donors (n = 4) were left untreated (empty circles) or were treated for 1 hr with increasing concentrations of zymosan, WGP, *A. fumigatus* germ tubes, or *C. albicans* yeasts (each at 2 × 10^5^/ml and 1 × 10^6^/ml). IL-1β synthesis in CD33^+^ cells was analyzed by intracellular cytokine stainings by flow cytometry. ^∗^p < 0.05 versus control/untreated conditions. Right panel: co-culture supernatants were collected after incubating isolated PBMCs (5 × 10^5^ cells/ml) with medium only (white bar), *A. fumigatus* germ tubes (1 × 10^5^ cells/ml), or *C. albicans* yeasts (1 × 10^5^/ml) for 3 days. IL-1β was quantified by ELISA. ^∗^p < 0.05 versus medium control conditions. (B) IL-1β signaling is involved in fungal-induced MDSC generation. Left panel: MDSCs were generated in vitro by incubating isolated PBMCs (5 × 10^5^ cells/ml) with *C. albicans* yeasts (1 × 10^5^/ml) or recombinant human IL-1β protein (0.01 μg/ml) for 6 days. ^∗^p < 0.05. Right panel: MDSCs (CD11b^+^Ly6G^+^) were quantified in spleens from *Il1r*^−/−^ and age-matched WT mice 2 days after i.v. infection with 1 × 10^5^ blastospores of *C. albicans*. ^∗^p < 0.05. (C) Fungal MDSC generation involves caspase-8. MDSCs were generated in vitro by incubating isolated PBMCs (5 × 10^5^ cells/ml) with *C. albicans* yeasts (1 × 10^5^/ml) for 6 days with or without pretreatment (where indicated) with the pan-caspase inhibitor Z-VAD-FMK (10 μM), the caspase-1 inhibitor Z-WEHD-FMK (50 μM), or the caspase-8 inhibitor Z-IETD-FMK (50 μM). IL-1β protein levels were quantified in cell culture supernatants by ELISA (note: two values were below detection limit). Caspase-8 activity was quantified in cell lysates using a luminescent assay. ^∗^p < 0.05. (D) Fungal MDSC-inducing capacity is ROS dependent. MDSCs were generated in vitro by incubating isolated PBMCs (5 × 10^5^ cells/ml) with different fungal morphotypes (1 × 10^5^ cells/ml) or zymosan (10 μg/ml) for 6 days. PBMCs were pretreated where indicated with the NADPH oxidase inhibitor DPI (0.1 μM) or the H_2_O_2_ converting enzyme catalase (100 U/l). ^∗^p < 0.05 blocking versus unblocked conditions. (E) Fungal MDSC induction in patients with ROS deficiency. Left panel: MDSCs were generated in vitro by incubating isolated PBMCs (5 × 10^5^ cells/ml) from healthy controls (n = 12) or patients with CGD (n = 3) with the Dectin-1/CARD9 ligands zymosan depleted (10 μg/ml) or dispersible WGP (20 μg/ml). Right panel: MDSCs were generated in vitro by incubating isolated PBMCs (5 × 10^5^ cells/ml) from healthy controls (n = 12) or CGD patients (n = 3) with different fungal morphotypes (1 × 10^5^ cells/ml) for 6 days. In (A)–(E) bars represent means ± SEM.

*C. albicans* induces interleukin-1 beta (IL-1β) in vitro (van de Veerdonk et al., 2009) and in vivo (Hise et al., 2009), which is critical for antifungal immunity (Vonk et al., 2006). Recent studies further provided evidence that IL-1β is involved in MDSC homeostasis (Bruchard et al., 2013). We observed an accumulation of intracellular IL-1β protein in CD33^+^ myeloid cells followed by IL-1β release upon Dectin-1 ligand- and fungal-driven MDSC induction (Figure 4A). IL-1β protein, in turn, was sufficient to drive MDSC generation to a comparable extent as *C. albicans* did (Figure 4B). Studies in *Il1r*^−/−^ mice, characterized by an increased susceptibility to *C. albicans* infection, demonstrated that abrogation of IL-1R signaling decreased MDSC accumulation in vivo (Figures 4B and S4A), and IL-1R antagonism in patients with autoinflammatory diseases decreased MDSCs (Figure S4B). As the inflammasome is the major mechanism driving IL-1β generation in myeloid cells through caspase activities, we blocked caspases chemically. We observed that pan-caspase inhibition largely abolished fungi-induced MDSC generation, which was not recapitulated by caspase-1 inhibition (Figure 4C). We therefore focused on caspase-8, since Dectin-1 activation was shown to trigger IL-1β processing by a caspase-8-dependent mechanism (Ganesan et al., 2014; Gringhuis et al., 2012). Indeed, fungal MDSC induction was paralleled by a substantial increase of caspase-8 activity, and caspase-8 inhibition diminished fungal-induced IL-1β production (Figure 4C) and the potential of fungi to induce MDSCs (Figure 4C). Conversely, supplementing IL-1β partially restored the abrogated MDSC generation upon caspase-8 inhibition (Figure S4C).

ROS are key factors in MDSC homeostasis (Gabrilovich and Nagaraj, 2009) and act downstream of Dectin-1 (Gross et al., 2009; Underhill et al., 2005). Therefore, we tested the involvement of ROS for fungal Dectin-1 ligand-induced MDSC generation using chemical inhibitors and cells from human CGD patients with ROS deficiency. These studies demonstrated that ROS contributed substantially to fungal MDSC induction (Figure 4D). Next, we investigated the interaction between ROS, caspase-8, and IL-1β and found that ROS inhibition dampened caspase-8 activity in response to fungi (Figure S4D). IL-1β, in turn, induced ROS production during MDSC culture, suggesting a positive feedback loop between caspase-8, IL-1β, and ROS in MDSC generation (Figures S4E and S4F).

## Discussion

While the complete genetic deletion of pro-inflammatory cytokines, particularly TNF-α, IL-1α/β, or IFN-γ, increases disease susceptibility in invasive fungal infections (Lionakis and Netea, 2013; Cheng et al., 2012; Gow et al., 2012; Netea et al., 2008, 2010), excessive inflammation causes collateral damage to the host (Carvalho et al., 2012; Romani et al., 2008), indicating that efficient protection against fungi requires a fine-tuned balance between pro-inflammatory effector and counter-regulatory immune mechanisms. Fungal infection induces an immunosuppressive state, and in murine models CD80^+^ neutrophilic cells have been shown to be importantly involved in this process (Mencacci et al., 2002; Romani, 2011; Romani et al., 1997). By combining human and murine experimental systems, we extend this concept by providing evidence for an MDSC-mediated mechanism by which fungi modulate host defense, orchestrated by Dectin-1/CARD9, ROS, caspase-8, and IL-1β. This effect seems to be specific for neutrophilic MDSCs, since monocytic MDSCs were unchanged under our experimental conditions and were previously found to be downregulated by β-glucans in tumor-bearing mice (Tian et al., 2013).

*C. albicans* and *A. fumigatus* infections differ substantially with respect to T cell dependency and organ manifestation (Garcia-Vidal et al., 2013). Our finding that neutrophilic MDSCs were protective in a murine model of systemic *C. albicans* infection, but had no effect on pulmonary *A. fumigatus* infection, underlines this disparity and suggests MDSCs as a potential therapeutic approach in invasive *C. albicans*, rather than *A. fumigatus* infections. The MDSC-mediated effect was associated with downregulated NK and T cell activation, and Th17 responses and supplementing IL-17A in vivo could, at least partially, dampen the protective effect of MDSCs. Based on previous studies showing that NK cells drive hyperinflammation in candidiasis in immunocompetent mice (Quintin et al., 2014) and that IL-17 promotes fungal survival (Zelante et al., 2012), we speculate that MDSCs in fungal infections could act beneficial for the host by dampening pathogenic hyperinflammatory NK and Th17 responses (Romani et al., 2008; Zelante et al., 2007). Accordingly, enhancing neutrophilic MDSCs may represent an anti-inflammatory treatment strategy for fungal infections, particularly with *C. albicans*.

Recent studies put the gut in the center of immunotolerance. Dectin-1 was found to control colitis and intestinal Th17 responses through sensing of the fungal mycobiome (Iliev et al., 2012). The immunological events downstream of Dectin-1 and their functional impact on Th17 cells remained elusive. Our results demonstrate that fungal Dectin-1/CARD9 signaling induces MDSCs to dampen T cell responses and suggest that the immune homeostasis in the gut could be modulated by fungal-induced MDSCs. Beyond fungi, the Dectin-1/CARD9 pathway has been involved in bacterial and viral infections (Hsu et al., 2007), suggesting that this mechanism could play a broader role in balancing inflammation at host-pathogen interfaces.

## Experimental Procedures

### Fungal Strains and Culture Conditions

*A. fumigatus* ATCC46645 conidia were incubated in RPMI at RT for 3 hr at 150 rpm to become swollen. Alternatively, conidia were cultured in RPMI overnight at RT, followed by germination in RPMI either at 37°C for 3 hr at 150 rpm to become germ tubes or at 37°C for 17 hr at 150 rpm to become hyphae. *C. albicans* SC5314 was grown on SAB agar plates at 25°C. One colony was inoculated and shaken at 200 rpm at 30°C in SAB broth overnight. To generate hyphae, live yeast forms of *C. albicans* were grown for 6 hr at 37°C in RPMI 1640. Killed yeasts and hyphae were prepared by heat treatment of the cell suspension at 95°C for 45 min or by fixing the cells for 1 hr with 4% paraformaldehyde followed by extensive washing with PBS to completely remove the fixing agent. The *C. albicans-*GFP *strain* TG6 was pre-cultured at 30°C, 200 rpm overnight in YPD medium.

### Generation, Isolation, and Characterization of MDSCs

Neutrophilic MDSCs in peripheral blood were quantified based on their lower density and surface marker profiles as published previously (Rieber et al., 2013). Human MDSCs were generated in vitro according to a published protocol (Lechner et al., 2010). Murine MDSCs were characterized by CD11b, Ly6G, and Ly6C. Flow cytometry was performed on a FACS Calibur (BD Biosciences). Human and murine MDSCs were isolated using MACS (MDSC Isolation Kit; Miltenyi Biotec).

### T Cell Suppression Assays

T cell suppression assays were performed as described previously (Rieber et al., 2013) using the CFSE method according to the manufacturer’s protocol (Invitrogen).

### Mouse Infection with *A. fumigatus* and *C. albicans*

Invasive *C. albicans* infection was established by IV injection in immunocompetent mice, whereas *A. fumigatus* infection was established by intranasal challenge in immunosuppressed mice. CD11b^+^Ly6G^+^ and CD11b^+^Ly6C^+^ cells in the spleens, BAL, and kidneys were quantified by FACS. For adoptive transfer experiments, CD11b^+^Ly6G^+^ MDSCs were isolated by MACS and transferred by IV injection of 4 or 5 × 10^6^ MDSCs per animal.

## Author Contributions

N.R. and D.H. designed the study, supervised experiments, performed analyses, and wrote the manuscript. H.Ö., A.S., and M.C. performed murine infection studies. A.S., S.N.K., M.O., M. Ballbach, Y.Z., and I.S. performed MDSC in vitro assays. M. Bouzani and J. Loeffler performed and supervised NK cell assays. J. Loeffler and S.K. provided fungi, contributed to the design of the study, and wrote the manuscript. J.A. and A.B. performed and analyzed murine infection studies. R.H., M.M., J. Loeffler, J. Liese, A.N.R.W., M.E., R.S., H.R.S., C.S., L.M., and B.G. co-designed the study, provided patient material, and wrote the manuscript. J.R. and G.D.B. provided mice and co-designed in vivo experiments.

## Supplemental Information

Document S1. Figures S1–S4 and Supplemental Experimental Procedures

Document S2. Article plus Supplemental Information
