# Supplementary material for: Pathogenic Fungi Regulate Immunity by Inducing Neutrophilic Myeloid-Derived Suppressor Cells
Source: Cell Host Microbe. 2015 Apr 8;17(4):507–14. doi: 10.1016/j.chom.2015.02.007 (PMC4400268; doi:10.1016/j.chom.2015.02.007)
Supplement: Document S1. Figures S1–S4 and Supplemental Experimental Procedures [file mmc1.pdf]

Cell Host & Microbe, Volume *17*

Supplemental Information

Pathogenic Fungi Regulate Immunity by Inducing Neutrophilic Myeloid-Derived Suppressor Cells

Nikolaus Rieber, Anurag Singh, Hasan Öz, Melanie Carevic, Maria Bouzani, Jorge Amich, Michael Ost, Zhiyong Ye, Marlene Ballbach, Iris Schäfer, Markus Mezger, Sascha N. Klimosch, Alexander N.R. Weber, Rupert Handgretinger, Sven Krappmann, Johannes Liese, Maik Engeholm, Rebecca Schüle, Helmut Rainer Salih, Laszlo Marodi, Carsten Speckmann, Bodo Grimbacher, Jürgen Ruland, Gordon D. Brown, Andreas Beilhack, Juergen Loeffler, and Dominik Hartl

Supplemental Data
Figure S1, related to Figure 1

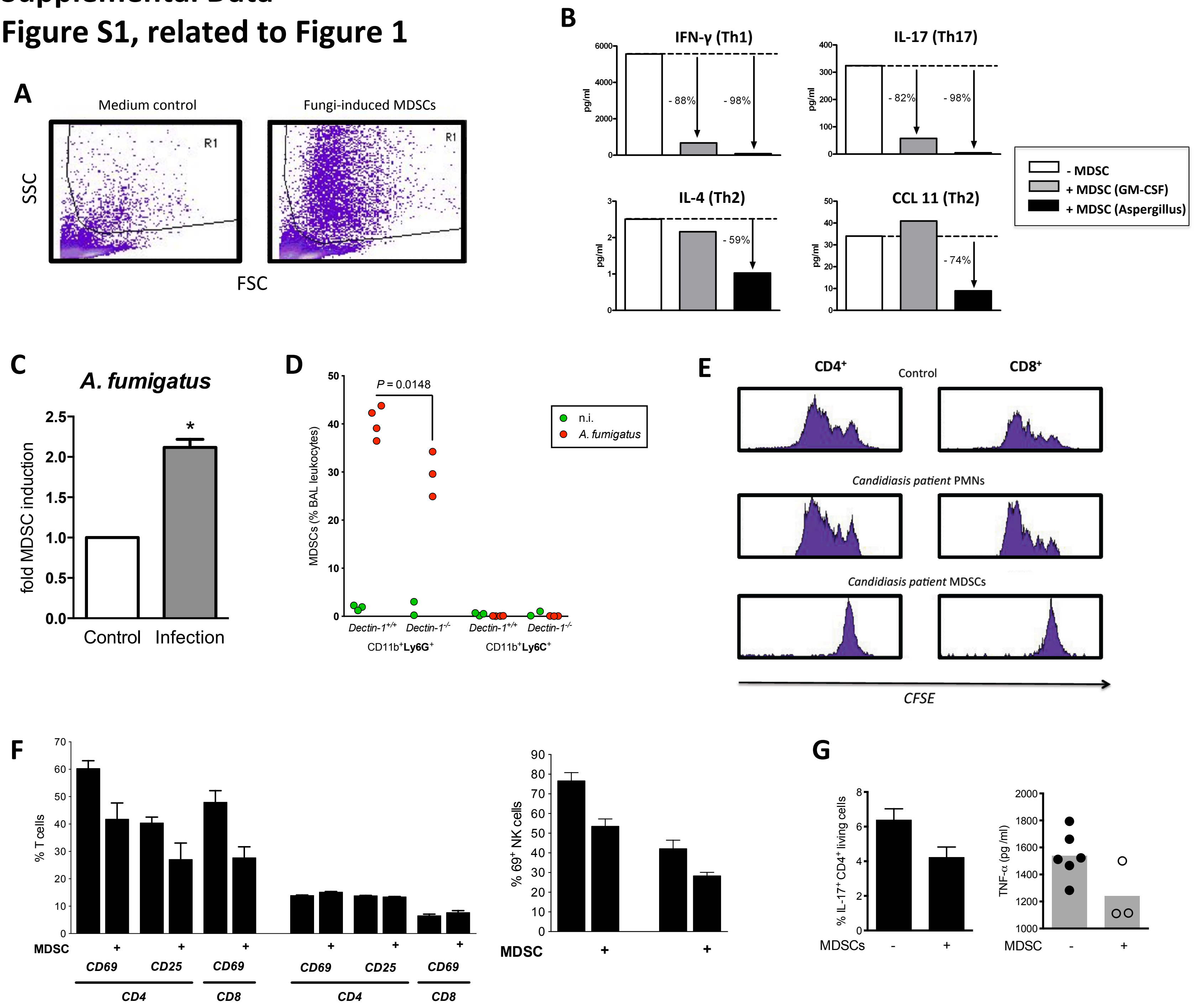

Figure S1. Phenotypic and functional MDSC characteristics

A. FSC/SSC characteristics of fungi-induced MDSCs *in vitro*
MDSCs were generated by incubating PBMCs (5 x 10^5 /ml) from healthy donors with medium only (negative control, 'Medium control') or *A. fumigatus* germ tubes (1 x 10^5 /ml, 'Fungi-induced MDSCs') for 6 days. Dot blots show representative MDSC gatings for subsequent immunophenotyping based on surface marker expression profiles as depicted in Figure 1a.

B. *Aspergillus*- and GM-CSF-induced MDSCs differentially affect Th1/Th17 and Th2 cytokine and chemokine levels
IL-2 and OKT-3 stimulated PBMCs were cultured in medium alone or together with Aspergillus- or GM-CSF- induced MDSCs for 96h. Cytokine and chemokine concentrations in culture supernatants were analyzed by multiplex array technology.

C. MDSC induction in immunodeficient mice
MDSC induction in immunodeficient mice: BALB/c wildtype mice were immunosuppressed with cyclophosphamide (150 mg/kg bw i.p.) and not infected (white bars) or challenged intranasally with 1x10^3 *A. fumigatus* conidia (grey bar) for three days. On the fourth day, $CD11b^+Ly6G^+$ MDSCs were quantified in lungs by FACS. The x-fold induction of $CD11b^+Ly6G^+$ MDSCs in the *A. fumigatus*–infected lung compared to control non-infected conditions is depicted. Bars represent means ± s.e.m. *P<0.05

D. MDSC induction in *Dectin-1*$^{-/-}$ mice
MDSC induction in *Dectin1*$^{-/-}$ mice: *Dectin-1*$^{-/-}$ mice and age-matched wildtype mice were challenged intranasally with 1x10^6 CFU *A. fumigatus* for three days. On the fourth day, a bronchoalveolar lavage was performed and granulocytic ($CD11b^+Ly6G^+$) or monocytic ($CD11b^+Ly6C^+$) MDSCs were quantified by flow cytometry.

E. *Candida*-induced neutrophilic MDSCs, but not conventional PMNs, suppress T-cell proliferation
Representative CFSE stainings, showing the effect of neutrophilic MDSCs or conventional autologous neutrophils (PMNs) isolated (MACS) from patients with invasive *C. albicans* infections on $CD4^+$ and $CD8^+$ T-cell proliferation.

F. Effect of MDSCs on inflammation
T- and NK-cell activation was quantified in kidneys (left bars) and spleens (right bars) (5 days p.i.) in the invasive *C. albicans* infection model with and without adoptive MDSC transfer. T and NK cell activation was measured by CD69 and CD25 surface expression on $CD4^+$ T cells, $CD8^+$ T cells and on $CD3^-DX5^+NKp46^+$ NK cells. T cell graph (left): the left bars show kidney, the right bars spleen. NK cell graph (right): the left bars show kidney, the right bars spleen.

G. IL-17A was stained intracellularly in $CD4^+$ splenocytes 5 days after adoptive MDSC transfer by flow cytometry. TNF-α protein levels were quantified in serum 5 days after adoptive MDSC transfer by Bioplex.

Figure S2,
related to Figure 1

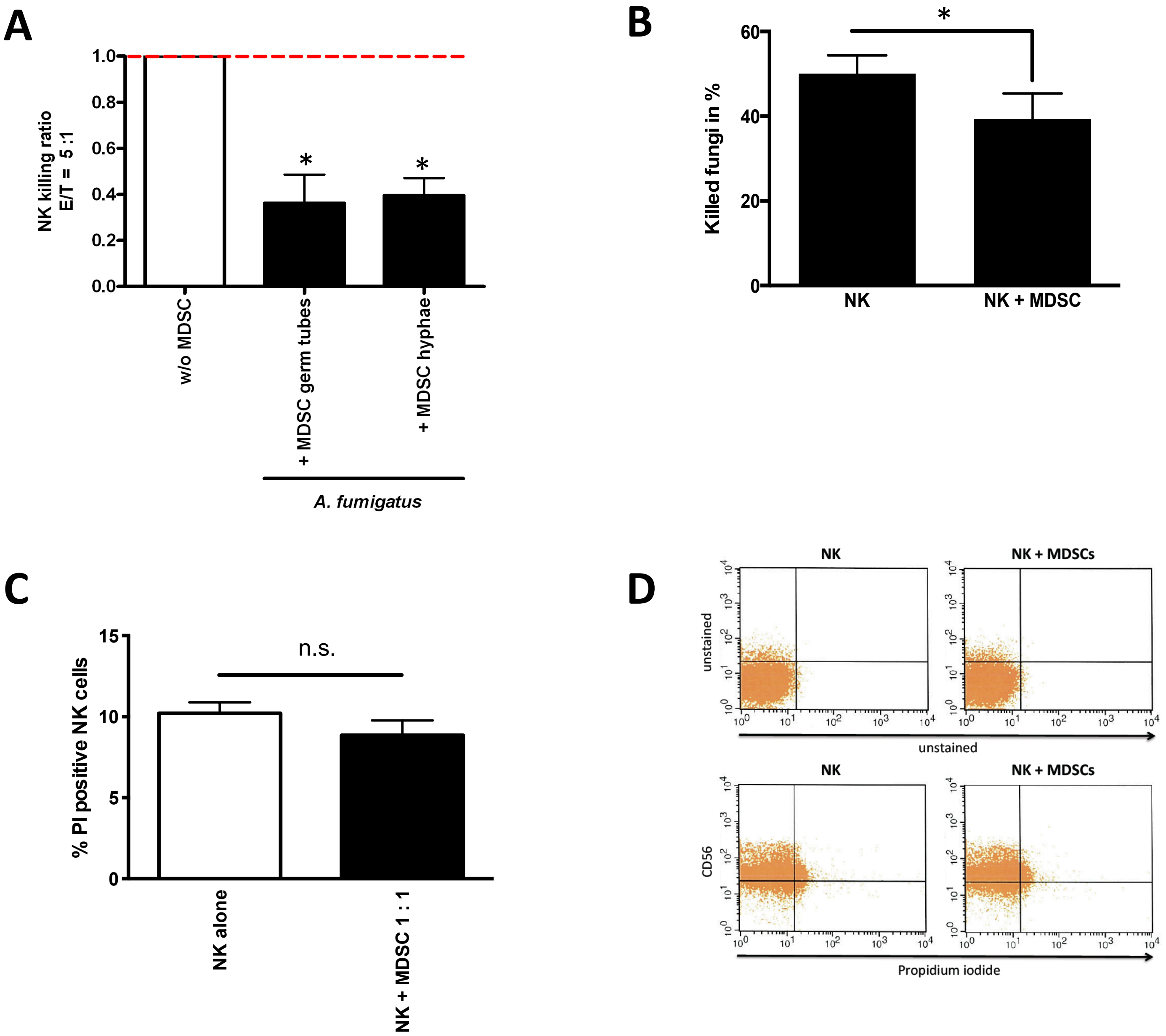

Figure S2: *Aspergillus*-induced MDSCs decrease antifungal NK killing activity

A. The suppressive effects of $CD33^{+}$-MACS-isolated MDSCs on NK cells were analyzed by measuring the NK cell cytotoxicity against K562 tumor cell line (europium release assay). MDSCs were generated by incubating PBMCs ($5x10^{5}$/ml) from healthy donors with *A. fumigatus* germ tubes ($1x10^{5}$/ml). MDSC to NK cell ratio was 1:1. NK (Effector, E) to K562 (Target, T) ratio was 5:1. Bars represent means ± s.e.m. *P<0.05;

B. Activated NK cells were co-cultured with purified MDSC at a 1:1 ratio for 16h. After co-culture, MDSC were depleted and purified NK cells were incubated with *A. fumigatus* germ tubes at a 1:1 ratio for 5h. Fungal cell viability was determined using an XTT assay. *P<0.05

C. MDSCs and NK cells were isolated from healthy PBMCs by magnetic bead technique. NK cells were cultured in medium alone or co-cultured together with MDSCs in a ratio of 1:1 overnight analogous to the cytotoxicity assays. Dead cells were stained with propidium iodide (PI). Bar graphs show percentages of PI positive dead NK cells within all NK cells. n.s. not significant

D. NK cell viability as assessed by propidium iodide staining for NK cells ($CD3^{-}CD56^{+}$ cells) in 1:1 NK-MDSC co-culture assays. The upper panel shows unstained controls from the same cells.

Figure S3, related to Figure 3

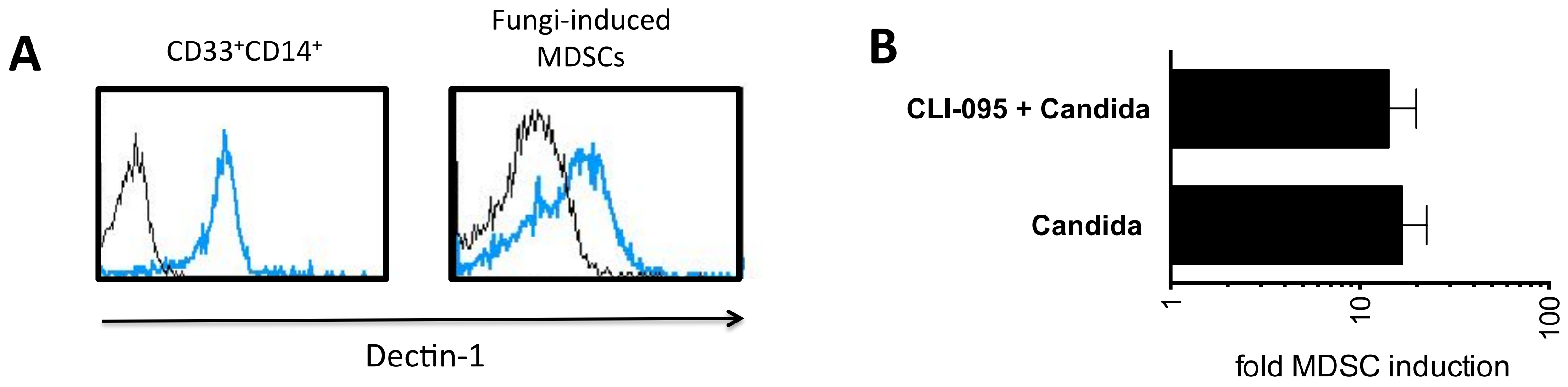

Figure S3: Dectin-1 and TLR4

A. Histograms show representative examples of Dectin-1 surface expression on fungi-induced MDSCs and $CD33^{+}CD14^{+}$ cells.

B. MDSCs were generated by incubating PBMCs ($5x10^5$/ml) from healthy donors with *C. albicans* (yeasts: $1x10^5$/ml) with or without 1h pretreatment with the TLR4 inhibitor CLI-095 (1µM). The x-fold induction of MDSCs compared to control conditions is depicted. Bars represent means ± s.e.m.

Figure S4, related to Figure 4

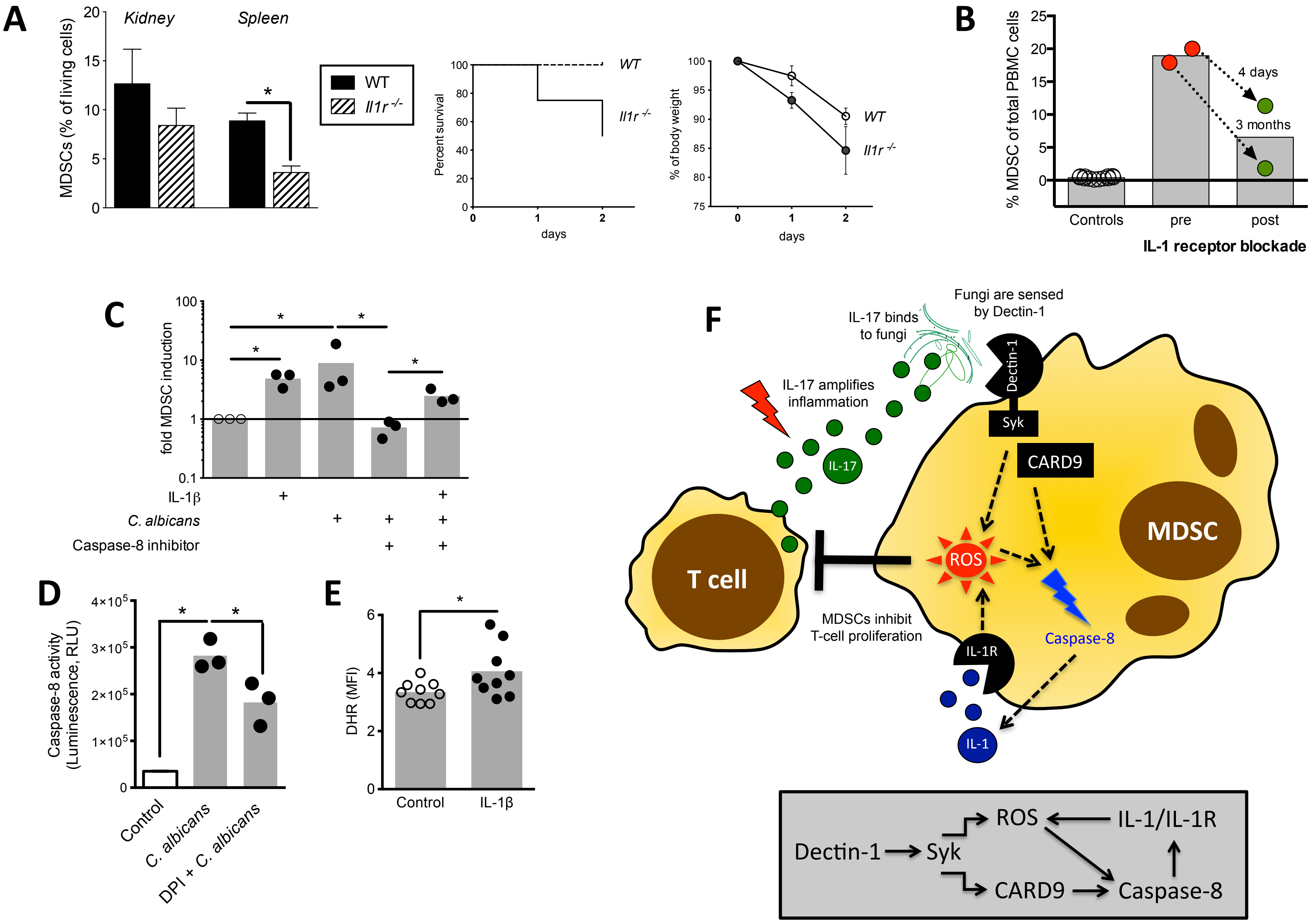

Figure S4: IL-1, Caspase-8 and ROS

A. WT and *Il1r*$^{-/-}$ mice were i.v. injected with 1×10^{5} blastospores of *Candida albicans* SC5314 per animal in 100 µl PBS. Mice were weighed daily and monitored for survival and weight loss. Mice with a weight loss of more than 20% or with serious symptoms of illness were euthanized. For MDSC quantification, mice were sacrificed and CD11b^{+}Ly6G^{+} MDSCs in the kidneys and spleens were quantified by FACS. Bars represent means ± s.e.m. **P*<0.05

B. MDSCs were quantified in peripheral blood of two patients before and after systemic anti-IL-1 therapy using the IL1-receptor-antagonist *anakinra* (3 mg/kg bw/d). Patient 1 (male, 2 years of age, 3 months on *anakinra*) had a severe chronic non-classified autoinflammatory disease and patient 2 (female, 9 years of age, 4 days on *anakinra*) suffered from systemic onset juvenile idiopathic arthritis (soJIA).

C. MDSCs were generated *in vitro* by incubating isolated PBMCs (5x10^{5} cells/ml) with *C. albicans* yeasts (1x10^{5}/ml) or recombinant human IL-1β (0.01 µg/ml) for 6 days with or without pretreatment with the caspase-8 inhibitor Z-IETD-FMK (50µM). MDSCs were quantified using flow cytometry. Bars represent means. **P*<0.05

D. MDSCs were generated *in vitro* by incubating isolated PBMCs (5x10^{5} cells/ml) with *C. albicans* yeasts (1x10^{5}/ml) for 6 days with or without pretreatment with the NADPH oxidase inhibitor DPI (0.1 µM). Caspase-8 activity was measured by a luminescent assay (Caspase-Glo 8 Assay from Promega, USA). Bars represent means. **P*<0.05

E. PBMCs of healthy donors were treated with medium only (control) or recombinant human IL-1β (0.1 µg/ml) for 4 hours. After stimulation with PMA (200nM) for another 8 minutes, ROS production was measured by DHR in CD33^{+} myeloid cells. Bars represent means. **P*<0.05

F. Proposed model of MDSC generation in invasive fungal infections: Fungal sensing through Dectin-1 triggers downstream signaling cascades involving Syk and CARD9, leading to caspase-8 activation. Caspase-8 drives interleukin-1 (IL-1) production. Released IL-1 binds to the IL-1 receptor (IL-1R) and enhances generation of ROS, which are essential for MDSC induction. Moreover, ROS are involved in fungal-driven caspase-8 activation. Generated MDSCs inhibit NK and T-cell responses, such as Th17 responses that amplify inflammation and may also directly affect fungal survival.

Supplemental Experimental Procedures

<u>Study subjects</u>

The study was conducted at the University Children's Hospital Tübingen (Germany). Informed consent was obtained from all subjects included in the study and all study methods were approved by the local ethics committee. At time of blood sampling all healthy subjects were without signs of infection, inflammation, or respiratory symptoms. Nine patients with invasive aspergillosis (positive *Aspergillus* galactomannan serum test and clinical signs of invasive aspergillosis) and six patients with invasive *Candida* bloodstream infections were also included in the study after written informed consent. These patients acquired invasive fungal infection during oncologic chemotherapy, after hematopoietic stem cell transplantation or showed fungal endocarditis. Moreover, five immunosuppressed patients after hematopoietic stem cell transplantation, but without fungal infections, were included as disease control group. In two patients with autoinflammatory diseases, MDSCs were quantified in peripheral blood before and after treatment with the IL-1R antagonist anakinra (3mg/kg/d). We further obtained blood from two patients with CARD9 deficiency and a medical history of several invasive fungal infections. The CARD9 mutations were: c.883G>A(hom) and c.883C>T(hom), both resulting in a premature termination codon (Q295X) consistent with a previously defined CARD9 defect (Glocker et al., 2009). On protein level no CARD9 protein could be detected in the patient's neutrophils and monocytes. We analyzed blood from a healthy subject with a homozygous Dectin-1 stop codon mutation (Tyr238X) consistent with a previously described Dectin-1 deficiency (Ferwerda et al., 2009). This mutation was identified through a whole exome sequencing approach for his affected daughter. In addition, we obtained fresh blood samples from three patients with chronic granulomatous disease (CGD) and complete ROS deficiency.

A. fumigatus strain and culture conditions

A. fumigatus ATCC46645 conidia were frozen at -80°C in glycerol stocks. After growing on Sab agar plates at 37°C, one colony was inoculated into Sab broth and shaken at 37°C overnight. Conidia were incubated in RPMI at room temperature (RT) for 3 h at 150 rpm to become swollen. Alternatively, conidia were cultured in RPMI overnight at RT, followed by germination in RPMI either at 37°C for 3 h at 150 rpm to become germ tubes or at 37°C for 17h at 150 rpm to become hyphae. Fungi were washed twice in PBS and heat-inactivated for 30 min at 95°C. Culture supernatants from conidia, germ tubes and hyphae were centrifuged at 8000 rpm for 15 min, followed by steril filtration using a 0.2 µm filter, respectively.

C. albicans strain and culture conditions

C. albicans SC5314 was stored as frozen stocks in 35 % glycerol at –80 °C and routinely grown on Sabouraud (Sab) agar plates at 25°C. One colony was inoculated and shaken at 200 rpm at 30°C in Sab broth (1% mycological peptone and 4% glucose) overnight. Cells were harvested by centrifugation and washed twice in Dulbecco's phosphate-buffered saline (PBS). Cells were counted in a haemocytometer and density was adjusted to the desired concentration in either PBS or RPMI 1640 medium. To generate hyphae, live yeast forms of *C. albicans* were grown for 6 h at 37°C in RPMI 1640 (Gibco-BRL). Killed yeasts and hyphae were prepared by heat treatment of the cell suspension at 95°C for 45 minutes or by fixing the cells for 1 h with 4% paraformaldehyde followed by extensive washing with PBS to completely remove the fixing agent. The *C. albicans*-GFP *strain* TG6 (a generous gift from Dr. Steffen Rupp, Fraunhofer IGB Stuttgart) was pre-cultured at 30°C, 200 rpm overnight in YPD medium. Cells were washed twice with sterile PBS and counted using a haemocytometer prior to use.

In vitro generation and isolation of human MDSCs

Human MDSCs were generated *in vitro* according to a previously published protocol (Lechner et al., 2010). Isolated human PBMCs were cultured in 12 well flat-bottom plates (Corning) or 25 cm² flasks (Greiner Bio-One) at 5 x 10^5 cells /ml in complete medium for 6 d, and GM-CSF (10 ng/ml, Genzyme), heat inactivated (95°C, 30min) *A. fumigatus* morphotypes (1:1 to 1:5 Aspergillus / PBMC ratio), *A. fumigatus* lysates (Miltenyi Biotec), *A. fumigatus* culture supernatants (4%), heat or formaldehyde inactivated *C. albicans* yeast and hyphae (1:5 to 1:20 Candida / PBMC ratio), curdlan (10 µg/ml, Invivogen), depleted zymosan (10 µg/ml, Invivogen) and WGP dispersible (20 µg/ml, Invivogen) were added as indicated in the respective figures. For blocking/inhibition experiments mouse anti-human Dectin-1 blocking antibody (15 µg/ml, AbD Serotec), WGP soluble (1 mg/ml, Invivogen), small molecule *syk*-inhibitor (100 nM, Calbiochem), the pan-caspase inhibitor Z-VAD-FMK (10µM, R&D Systems), the caspase-8 inhibitor Z-IETD-FMK (50µM, R&D Systems), the caspase-1 inhibitor Z-WEHD-FMK (50µM, R&D Systems), DPI (0.1µM, Sigma-Aldrich), Catalase (100 U/l, Sigma-Aldrich), the TLR4 inhibitor CLI-095 (1µM, Invivogen) and/or cytochalasin D (2µg/ml; Enzo Life Sciences) were added as indicated in the respective figures. PBMCs cultured in medium alone were run in parallel as a control for each experiment. Medium and supplements were refreshed after three days. After six days, all cells were collected from PBMC cultures. Adherent cells were removed using non-protease cell detachment solution Detachin (Genlantis). MDSCs were characterized as $CD33^+CD11b^+CD16^+CD14^-$ cells using recently established species-specific MDSC markers (Rieber et al., 2013a; Rieber et al., 2013b). For functional studies $CD33^+$ MDSCs were isolated from each culture using anti-CD33 magnetic microbeads and LS column separation (Miltenyi Biotech) with two sequential separation steps according to manufacturer's instructions.

Flow cytometry

Neutrophilic MDSCs in peripheral blood were quantified as published previously by our group (Rieber et al., 2013a). Antibodies against human CD3, CD4, CD8, CD14, CD16, CD66b, HLA-DR and CXCR4 were purchased from BD Pharmingen. Antibodies against CD11b and CD33 were purchased from MiltenyiBiotec. Antibodies against Dectin-1 were purchased from R&D Systems. Mouse IgG1-FITC, Mouse IgM-FITC, Mouse IgG1-PE and Mouse IgG1-APC (BD Pharmingen) were used as isotype controls. Antibodies against mouse CD11b, Ly6G and Ly6C were from BD Biosciences, anti-mouse CXCR4 was from Biolegend. Anti-mouse CD4 and IL-17A were from Miltenyi Biotech. CD3, CD8, CD25, CD69, NKp46, DX5 and the corresponding isotype controls were from Biolegend. T cells were characterized by CD3, CD4, CD8, CD25 and CD69 stainings. NK were characterized by $CD3^-$, NKp46, DX5 and CD69 stainings. Where indicated, T- and NK- cell activation in mice were analyzed in spleen and kidney tissues. Leukocyte enrichment/isolation from kidney tissues was performed as described previously (Lionakis et al., 2011). In brief, kidneys were aseptically removed, finely minced and digested with Liberase TL and DNase (Roche) for 30 min with intermittent shaking at 37°C. Digested tissue was passed through a 70-μm filter, washed with sterile PBS and remaining red cells were lysed with lysis buffer. Resulting suspensions were passed through a 40-μm filter and washed with PBS. Pellet was resuspended in 8 ml of 40% Percoll (GE Healthcare). Leukocyte enrichment was performed by overlaying Percoll-cell suspension on 3 ml of 70% Percoll solution, and centrifugation at 2,000 rpm without brakes for 30 min at RT. The interphase was collected carefully, washed in PBS and suspended in FACS buffer. Cells were counted using a haematocytometer. Flow cytometry was performed on a FACS Calibur (BD). Results were expressed as percent of positive cells and mean fluorescence intensity (MFI). Calculations were performed with BD CellQuestPro analysis software.

T-cell suppression assays

T-cell suppression assays were performed as described previously by us in detail (Rieber et al., 2013a). Responder-PBMCs were obtained from healthy volunteers and stained with carboxyfluoresceinsuccinimidyl ester (CFSE) according to the manufacturer's protocol (Invitrogen). PBMCs were stimulated with 100 U/ml Interleukin-2 (IL-2; R&D Systems) and 1 µg/ml OKT3 (Janssen Cilag). In a standardized way, 60,000 PBMCs per well in RPMI1640 (Biochrom) were seeded in a 96-well microtitre plate and RPMI1640 only or 3,750 (1:16) to 30,000 (1:2) MDSCs in RPMI1640 were added. The cell culture was supplemented with 10% heat-inactivated human serum, 2mM glutamine, 100 IU/ml penicillin, and 100 mg/ml streptomycin. After 96h of incubation in a humidified atmosphere at 37°C and 5% CO_2 cells were harvested and supernatants were frozen in -20°C. For mouse T-cell suppression assays, $CD11b^+Ly6G^+$ MDSCs were isolated from bone-marrows using MACS (MDSC isolation kit, Miltenyi Biotec, Germany) and were co-cultured for three days (37°C, 5% CO_2) with T cells ($CD4^+$ splenocytes) at a 1:2 (MDSCs : T-cell) ratio. T cells were activated with CD3/CD28-beads (mouse T cell activation kit, Miltenyi Biotec, Germany) and recombinant mouse IL-2 (50 U/ml, Biolegend). The cell culture was supplemented with 10% fetal bovine serum and 2mM glutamine. CFSE-fluorescence intensity for human and murine assays was analyzed by flow cytometry to determine polyclonal T- cell proliferation.

Intracellular cytokine analysis

Erythrocytes were lysed with Pharm Lyse Buffer (BD Pharmingen), leukocytes were washed with cold PBS and resuspended in RPMI (3 ml) with supplements (10% human serum, 100 U/ml Penicillin, 100 U/ml Streptomycin, 2 mM L-glutamine; Gibco) with the addition of benzonase (50 U/ml; Promega). The cells were plated into a 96-well flat bottom plate (200 µl), stimulated as indicated and were cultured for one hour (37°C; 5% CO_2). Brefeldin A (Sigma) was added (c_{final} = 10 µg/ml) and cells were cultured overnight. The cells were harvested and washed with cold PBS (0.1% sodium azide). LIVE⁄DEAD Fixable Aqua was used to stain dead cells (Life Technologies). Fc-receptors were blocked with Flebogamma (50 µg/ml, Grifols Biologicals) and cells were stained extracellularly with anti-CD33 PerCP-Cy5.5 (BD Pharmingen). The cells were fixed and permeabilized with Cytofix/ Cytoperm (BD Pharmingen), Fc-receptors were blocked as before and IL-1β was stained intracellularly (eBioscience). Flow cytometry was performed on a FACS Canto II (BD Pharmingen). Results were expressed as percent of $CD33^+IL\text{-}1\beta^+$ cells. In murine infections models, IL-17A was stained intracellularly in $CD4^+$ splenocytes by flow cytometry as described previously by us (Mays et al., 2013). Calculations were performed with FlowJo analysis software (Tree Star).

Cytokine and Caspase analyses

IL-1β ELISA Kits (R&D systems) were used to quantify cytokine protein levels. Multiplex cytokine array analyses in human MDSC / PBMC co-culture supernatants and mouse serum were performed using human and mouse Bioplex protein multi-array systems (Bio-Rad). Caspase-8 activity in cell lysates was analysed using a luminescent assay (Caspase-Glo 8 Assay from Promega, USA). Assays were performed according to the manufacturer's recommendations.

NK cell cytotoxicity assay

NK cell cytotoxicity assays were performed as described previously by us (Rieber et al., 2013b). In brief, NK cells were separated by MACS and co-incubated with MDSCs for 16h in a 1:1 ratio. Afterwards cytolytic activity of NK cells against K562 tumor cell line was tested in a BATDA europium release. E:T ratio was 5:1. We used the ratios of NK cell cytotoxicity in the presence of MDSCs / NK cell cytotoxicity without MDSCs for statistical analysis. NK cell cytotoxicity without MDSCs was set to a fixed value of 1. For *A. fumigatus* killing, NK cells were pre-stimulated with 1000 IU IL-2 (MiltenyiBiotec) for 24h. Activated NK cells were co-cultured with purified MDSC at a 1:1 ratio for 16h. After co-culture, MDSC were depleted using a MACS separation column (MiltenyiBiotec) and purified NK cells were incubated with *A. fumigatus* germ tubes at a 1:1 ratio for 5h. NK cells were lysed using ddH2O and a cell viability assay (2,3-Bis-(2-methoxy-4-nitro-5-sulfophenyl)-2H-tetrazolium-5-carboxanilid [XTT] assay) was performed to determine killing of *A. fumigatus*. Propidium iodide was used to analyse NK cell death.

Fungal phagocytosis and killing assays

MDSCs were isolated by MACS separation and the phagocyte killing assay was performed as described previously for neutrophils (Bambach et al., 2009). Briefly, 1 × 10^6 MDSC were cocultured with 1 × 10^5 serum opsonized *C. albicans* (10:1 ratio) for 3 h at 37°C in RPMI. The cells were centrifuged, and suspended in sterile water for lysis. Serial dilutions were performed of the cell suspension and 100 µl was plated onto YPD agar plates containing penicillin and streptomycin. Plates were incubated for 24-48 h at 37°C and CFUs were enumerated. The phagocytic capacity of human and murine MDSCs was further assessed by FACS. Therefore, MACS-isolated human granulocytic MDSCs (low density $CD66b^{+}CD33^{+}$ cells) were co-cultured with GFP–labelled *C. albicans* spores (MOI=1) in RPMI medium at 37 °C for 90 min. MACS-isolated mouse granulocytic $CD11b^{+}Ly6G^{+}$ MDSCs were co-cultured with GFP–labelled *C. albicans* spores (MOI=4) in RPMI medium at 37°C for 90 minutes. GFP expression of MDSCs was analyzed by FACS.

Mouse infection with *A. fumigatus* and *C. albicans*

All animal studies were approved by the local authorities (TVA/RP IDs: AZ 35/9185.81-2 / K5/13). *A. fumigatus* conidia (strain ATCC46645) were harvested on the day of infection, submerged in 0.9% NaCl + 0.002% Tween-20, filtered, centrifuged for 10 min 3000 rpm and resuspended in 5 mL 0.9% NaCl + 0.002% Tween-20. *Card9*$^{-/-}$ mice on a C57/BL6 background, *Dectin-1*$^{-/-}$ mice on a BALB/c background or age-matched C57/BL6 or BALB/c WT mice, respectively, were challenged intranasally with $1x10^4$ or $1x10^6$ *A. fumigatus* conidia for three days. At the fourth day, a bronchoalveolar lavage (BAL) was performed and $CD11b^+Ly6G^+$ and $CD11b^+Ly6C^+$ cells were quantified in BAL fluid by FACS. *C. albicans* (strain SC5314) was grown at 30°C overnight in liquid YPD (yeast extract, peptone, and dextrose) medium containing penicillin and streptomycin. Cells were collected by centrifugation, washed and resuspended in PBS. Required cell density was adjusted using a haemocytometer. For infection, female C57BL/6 mice were injected via the lateral tail vein with 2.5×10^5 or 5×10^5 blastospores per animal in 200 µl PBS. Control animals were given PBS only. $CD11b^+Ly6G^+$ cells in the spleens were quantified by FACS. Where indicated, *C. albicans* infection experiments (see details above) were performed in *Il1r*$^{-/-}$ on a C57BL/6 background and matched C57BL/6 WT mice. For adoptive transfer experiments, $CD11b^+Ly6G^+$ MDSCs were isolated from the bone marrow of healthy female *BALB/c* mice by MACS (MDSC isolation kit, Miltenyi Biotec, Germany). Transfer was performed by injecting $4\text{-}5\times10^6$ MDSCs per animal into eight to twelve weeks old (18–22 g) female *BALB/c* mice via lateral tail vein. Two hours after the MDSC transfer, mice were i.v. injected with 1×10^5 blastospores of *C. albicans* (SC5314 in 100 µl PBS). Mice were weighed daily and monitored for survival and signs of morbidity. Mice with a weight loss of more than 20% were euthanized. For CFU determination, mice were euthanized at day 5 post-infection. The kidneys were aseptically removed, homogenized in 1ml PBS, serially diluted, and plated in duplicate on YPD agar containing penicillin and streptomycin. CFUs were determined after 48 hrs of incubation at 37°C. To assess the impact of phagocytosis *in vivo*, MDSCs were pretreated with Cytochalasin D (1µg/ml, Enzo Life Sciences) prior to adoptive transfer. Where indicated, recombinant mouse IL-17A protein (Biolegend) was mixed with *C. albicans* suspension and injected via mouse tail vein (5µg IL-17A protein/mouse). For invasive pulmonary *A. fumigatus* infection studies, eight to twelve weeks old (18–22 g) female *BALB/c* mice were immunosuppressed by treatment with cyclophosphamide (150 mg/kg bw i.p., days -3 and -1). Mice where challenged intranasally with $1x10^3$ or $2x10^5$ *A. fumigatus* conidia (freshly harvested from three days old plates). For survival studies, mice were challenged once with *A. fumigatus*, for MDSC induction studies for three consecutive days, as indicated in the respective figure legends. Where indicated, MDSC transfer was performed by intravenous injection of 4×10^6 MDSCs per animal prior to infection and mice were monitored for survival as described above.

Statistical analysis

Statistical analysis was done using GraphPad Prism 5.0 (Graph Pad Software). Differences between the groups were determined by Students' t test. Survival was calculated using the Log-rank (Mantel-Cox) test. A *P* value of <0.05 was considered to be significant.

Supplemental References

Bambach, A., Fernandes, M.P., Ghosh, A., Kruppa, M., Alex, D., Li, D., Fonzi, W.A., Chauhan, N., Sun, N., Agrellos, O.A.*, et al.* (2009). Goa1p of Candida albicans localizes to the mitochondria during stress and is required for mitochondrial function and virulence. Eukaryotic cell *8*, 1706-1720.

Ferwerda, B., Ferwerda, G., Plantinga, T.S., Willment, J.A., van Spriel, A.B., Venselaar, H., Elbers, C.C., Johnson, M.D., Cambi, A., Huysamen, C.*, et al.* (2009). Human dectin-1 deficiency and mucocutaneous fungal infections. N Engl J Med *361*, 1760-1767.

Glocker, E.O., Hennigs, A., Nabavi, M., Schaffer, A.A., Woellner, C., Salzer, U., Pfeifer, D., Veelken, H., Warnatz, K., Tahami, F.*, et al.* (2009). A Homozygous CARD9 Mutation in a Family with Susceptibility to Fungal Infections. New Engl J Med *361*, 1727-1735.

Lechner, M.G., Liebertz, D.J., and Epstein, A.L. (2010). Characterization of cytokine-induced myeloid-derived suppressor cells from normal human peripheral blood mononuclear cells. J Immunol *185*, 2273-2284.

Mays, L.E., Ammon-Treiber, S., Mothes, B., Alkhaled, M., Rottenberger, J., Muller-Hermelink, E.S., Grimm, M., Mezger, M., Beer-Hammer, S., von Stebut, E.*, et al.* (2013). Modified Foxp3 mRNA protects against asthma through an IL-10-dependent mechanism. J Clin Invest *123*, 1216-1228.

Rieber, N., Brand, A., Hector, A., Graepler-Mainka, U., Ost, M., Schafer, I., Wecker, I., Neri, D., Wirth, A., Mays, L.*, et al.* (2013a). Flagellin Induces Myeloid-Derived Suppressor Cells: Implications for Pseudomonas aeruginosa Infection in Cystic Fibrosis Lung Disease. J Immunol *190*, 1276-1284.

Rieber, N., Gille, C., Kostlin, N., Schafer, I., Spring, B., Ost, M., Spieles, H., Kugel, H.A., Pfeiffer, M., Heininger, V.*, et al.* (2013b). Neutrophilic myeloid-derived suppressor cells in cord blood modulate innate and adaptive immune responses. Clin Exp Immunol *174*, 45-52.
