# Supplementary material for: Pathogenic Fungi Regulate Immunity by Inducing Neutrophilic Myeloid-Derived Suppressor Cells
Source: Cell Host Microbe. 2015 Apr 8;17(4):507–14. doi: 10.1016/j.chom.2015.02.007 (PMC4400268; doi:10.1016/j.chom.2015.02.007)
Supplement: Document S2. Article plus Supplemental Information [file mmc2.pdf]

Short Article

Cell Host & Microbe

Pathogenic Fungi Regulate Immunity by Inducing Neutrophilic Myeloid-Derived Suppressor Cells

Graphical Abstract

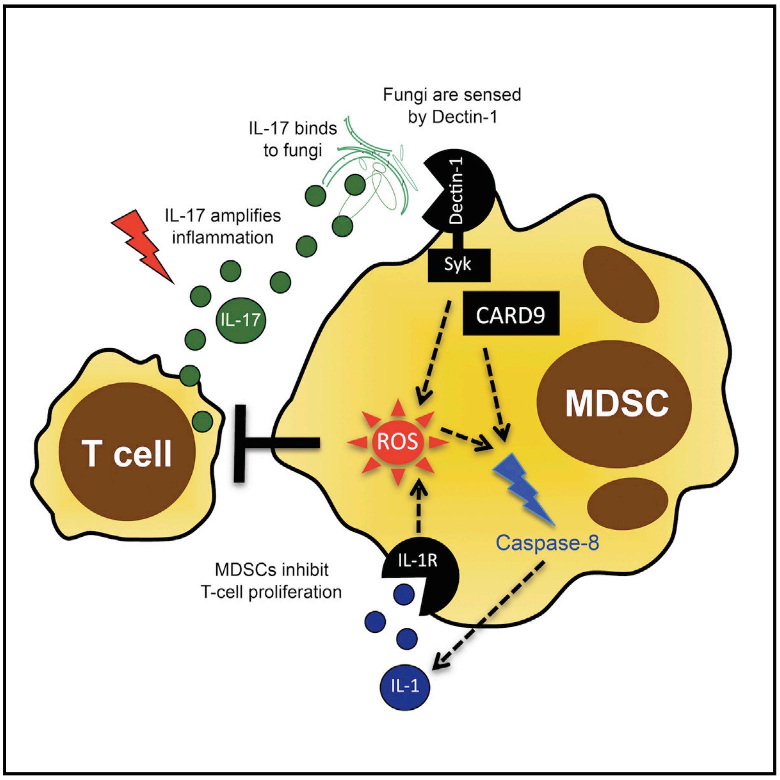

Authors

Nikolaus Rieber, Anurag Singh, ..., Juergen Loeffler, Dominik Hartl

Correspondence

nikolaus.rieber@med.uni-tuebingen.de (N.R.),
dominik.hartl@med.uni-tuebingen.de (D.H.)

In Brief

Myeloid-derived suppressor cells (MDSCs) are innate immune cells that suppress T cell responses. Rieber et al. show that pathogenic fungi *Aspergillus fumigatus* and *Candida albicans* induce MDSCs through mechanisms involving Dectin-1/CARD as well as downstream ROS and IL-1β production, and that transfer of MDSCs protects against invasive *Candida* infection.

Highlights

- Pathogenic fungi induce myeloid-derived suppressor cells (MDSCs)
- MDSC induction involves Dectin-1/CARD9, ROS, caspase-8, and IL-1
- MDSCs dampen T and NK cell immune responses
- Adoptive transfer of MDSCs improves survival in *Candida* infection in vivo

Rieber et al., 2015, Cell Host & Microbe *17*, 507–514
April 8, 2015 ©2015 The Authors
http://dx.doi.org/10.1016/j.chom.2015.02.007

Cell Host & Microbe
Short Article

CellPress

Pathogenic Fungi Regulate Immunity by Inducing Neutrophilic Myeloid-Derived Suppressor Cells

Nikolaus Rieber,[1,*] Anurag Singh,[1] Hasan Öz,[1] Melanie Carevic,[1] Maria Bouzani,[2] Jorge Amich,[3] Michael Ost,[1] Zhiyong Ye,[1,4] Marlene Ballbach,[1] Iris Schäfer,[1] Markus Mezger,[1] Sascha N. Klimosch,[5] Alexander N.R. Weber,[5] Rupert Handgretinger,[1] Sven Krappmann,[6] Johannes Liese,[7] Maik Engeholm,[8] Rebecca Schüle,[8] Helmut Rainer Salih,[9] Laszlo Marodi,[10] Carsten Speckmann,[11] Bodo Grimbacher,[11] Jürgen Ruland,[12] Gordon D. Brown,[13] Andreas Beilhack,[3] Juergen Loeffler,[2] and Dominik Hartl[1,*]
[1]Department of Pediatrics I, University of Tübingen, 72076 Tübingen, Germany
[2]Department of Medicine II, University of Würzburg, 97080 Würzburg, Germany
[3]IZKF Research Group for Experimental Stem Cell Transplantation, Department of Medicine II, 97080 Würzburg, Germany
[4]Department of Pediatrics, Yong Loo Lin School of Medicine, National University of Singapore, Singapore 119077, Singapore
[5]Institute of Cell Biology, Department of Immunology, University of Tübingen, 72076 Tübingen, Germany
[6]Microbiology Institute – Clinical Microbiology, Immunology and Hygiene, University Hospital of Erlangen and Friedrich-Alexander University Erlangen-Nürnberg, 91054 Erlangen, Germany
[7]Department of Pediatrics, University of Würzburg, 97080 Würzburg, Germany
[8]Department of Neurology
[9]Department of Oncology
University of Tübingen, 72076 Tübingen, Germany
[10]Department of Infectious and Pediatric Immunology, Medical and Health Science Center, University of Debrecen, 4032 Debrecen, Hungary
[11]Centre of Chronic Immunodeficiency (CCI), University Medical Center Freiburg and University of Freiburg, 79106 Freiburg, Germany
[12]Institut für Klinische Chemie und Pathobiochemie, Klinikum rechts der Isar, Technische Universität München, 81675 Munich, Germany
[13]Aberdeen Fungal Group, Section of Immunology and Infection, University of Aberdeen, AB24 3FX Aberdeen, UK
*Correspondence: nikolaus.rieber@med.uni-tuebingen.de (N.R.), dominik.hartl@med.uni-tuebingen.de (D.H.)
http://dx.doi.org/10.1016/j.chom.2015.02.007
This is an open access article under the CC BY license (http://creativecommons.org/licenses/by/4.0/).

SUMMARY

Despite continuous contact with fungi, immunocompetent individuals rarely develop pro-inflammatory antifungal immune responses. The underlying tolerogenic mechanisms are incompletely understood. Using both mouse models and human patients, we show that infection with the human pathogenic fungi *Aspergillus fumigatus* and *Candida albicans* induces a distinct subset of neutrophilic myeloid-derived suppressor cells (MDSCs), which functionally suppress T and NK cell responses. Mechanistically, pathogenic fungi induce neutrophilic MDSCs through the pattern recognition receptor Dectin-1 and its downstream adaptor protein CARD9. Fungal MDSC induction is further dependent on pathways downstream of Dectin-1 signaling, notably reactive oxygen species (ROS) generation as well as caspase-8 activity and interleukin-1 (IL-1) production. Additionally, exogenous IL-1β induces MDSCs to comparable levels observed during *C. albicans* infection. Adoptive transfer and survival experiments show that MDSCs are protective during invasive *C. albicans* infection, but not *A. fumigatus* infection. These studies define an innate immune mechanism by which pathogenic fungi regulate host defense.

INTRODUCTION

At mucosal sites, the human immune system is faced continuously with microbes, rendering fine-tuned immune responses essential to protect against pathogenic, while maintaining tolerance against harmless, species. This immune balance is of particular relevance for fungi, inhaled daily as spores or present in the gut microflora as commensal yeasts (Romani, 2011). While immunocompetent individuals do not develop invasive fungal infections, infections are a major problem in patients undergoing immunosuppression, for instance, at solid organ or hematopoietic stem cell transplantation (Garcia-Vidal et al., 2013).

Fungi are recognized through pattern recognition receptors, mainly C-type lectin receptors (with Dectin-1 as the prototypic one) (Steele et al., 2005), toll-like receptors (TLRs), and pentraxin 3 (PTX3) (Garlanda et al., 2002; Werner et al., 2009). A certain level of inflammation is essential to control fungal infections (Brown, 2010), but hyperinflammatory responses seem to cause more harm than good to the host. Particularly, Th17-driven hyperinflammatory responses have been shown to promote fungal growth (Zelante et al., 2012), to impair fungal clearance, and to drive tissue damage (Romani et al., 2008; Zelante et al., 2007). Generation of reactive oxygen species (ROS), indoleamine 2,3-dioxygenase (IDO) activity, and activation of the TIR domain-containing adaptor-inducing interferon-β (TRIF) pathway were found to limit hyperinflammatory responses toward *Aspergillus fumigatus* (Romani, 2011; Romani et al., 2009). Yet, the cellular mechanisms by which fungi

control T cell activation and maintain tolerogenic host-pathogen bistability remain incompletely understood.

Myeloid-derived suppressor cells (MDSCs) are innate immune cells characterized by their capacity to suppress T cell responses (Gabrilovich and Nagaraj, 2009). MDSCs comprise a neutrophilic and a monocytic subset. While the functional impact of MDSCs in cancer is established, their role in host-pathogen interactions is poorly defined. We hypothesized that fungal infections induce MDSCs that modulate disease outcome.

RESULTS

We analyzed the effect of the human-pathogenic fungi *A. fumigatus* and *C. albicans* on human immune cells and noticed the appearance of a cell population that was different from monocytes ($CD14^{-}$), and expressed the myeloid markers $CD33^{+}$, $CD11b^{+}$, $CD16^{+}$, and CXCR4 (Figures 1A and S1A). Fungi-induced myeloid cells strongly suppressed both $CD4^{+}$ and $CD8^{+}$ T cell proliferation in a dose-dependent manner (Figure 1B), which defines MDSCs. Fungi-induced MDSCs also suppressed innate natural killer (NK) cell responses, without affecting cell survival (Figure S2). In contrast to growth factor-induced MDSCs, fungi-induced MDSCs dampened Th2 responses, which play essential roles in fungal asthma (Kreindler et al., 2010) (Figure S1B). We quantified MDSCs in patients with invasive fungal infections and challenged mice with *A. fumigatus* or *C. albicans*. MDSCs accumulated in both *A. fumigatus*- and *C. albicans*-infected patients compared to healthy and disease control patients without fungal infections (Figure 1C). Murine studies further showed that systemic or pulmonary fungal challenge with *C. albicans* (invasive disseminated candidiasis) or *A. fumigatus* (pulmonary aspergillosis), as the clinically relevant routes of infection, dose-dependently triggered the recruitment of MDSCs in both immunocompetent and immunosuppressed conditions, with a stronger MDSC induction seen in immunocompetent animals (Figures 1D and S1C). MDSCs expressed neutrophilic markers in both man and mice, resembling the neutrophilic subtype of MDSCs (Rieber et al., 2013), while monocytic MDSC subsets were not induced (Figure S1D). Fungi-induced MDSCs functionally suppressed T cell proliferation (Figure 1C), while autologous conventional neutrophils failed to do (Figure S1E).

We adoptively transferred T cell-suppressive neutrophilic MDSCs and monitored their impact on survival in fungal infection. While a single dose of adoptively transferred MDSCs was protective in systemic *C. albicans* infection, MDSCs had no impact on *A. fumigatus* infection (Figure 1E). Septic shock determines mortality in candidiasis (Spellberg et al., 2005), and the interplay of fungal growth and renal immunopathology was shown to correlate with host survival (Lionakis et al., 2011, 2013; Lionakis and Netea, 2013; Spellberg et al., 2003). Adoptively transferred MDSCs dampened renal T and NK cell activation and systemic Th17 and TNF-α cytokine responses (Figures S1F and S1G). Conversely, supplementing IL-17A dampened the MDSC-mediated protective effect (Figure 2A). Besides these immunomodulatory effects, MDSCs might also act directly antifungal, as our in vitro studies showed that they can phagocytose and kill fungi (Figure 2B). However, direct antifungal effects could hardly explain the beneficial effect of MDSCs in candidiasis: (i) adoptively transferred MDSCs had no effect on fungal burden in vivo (Figure 2A), (ii) inhibition of phagocytosis only partially diminished the protection conferred by MDSCs (Figure 2A), and (iii) MDSCs were exclusively protective in immunocompetent mice (*C. albicans* infection model), with no effect in immunosuppressed (neutropenic) mice (*A. fumigatus* infection model).

The potency of *A. fumigatus* to induce MDSCs was most pronounced for germ tubes and hyphae, morphotypes characteristic for invasive fungal infections (Figure 1A) (Aimanianda et al., 2009; Hohl et al., 2005; Moyes et al., 2010). The MDSC-inducing fungal factor was present in conditioned supernatants and was heat resistant (Figure 3A), pointing to β-glucans as the bioactive component. We therefore focused on Dectin-1 as β-glucan receptor and key fungal sensing system in myeloid cells. Fungi-induced MDSCs expressed Dectin-1, and blocking Dectin-1 prior to fungal exposure diminished the MDSC-inducing effect, while blocking of TLR 4 had no effect (Figures 3B and S3). Furthermore, Dectin-1 receptor activation mimicked the generation of neutrophilic MDSCs phenotypically and functionally (Figures 3C and 3D). Dectin-1 receptor signaling was confirmed by blocking of the spleen tyrosine kinase Syk, which acts downstream of Dectin-1 (Figure 3B). We further used cells from human genetic Dectin-1 deficiency and used *Dectin-1* knockout mice for fungal infection models. The potential of fungi or fungal patterns to induce neutrophilic MDSCs was diminished in human and, albeit to a lesser extent, murine Dectin-1 deficiency (Figures 3E and S1D). We analyzed the role of caspase recruitment domain 9 (CARD9), a downstream adaptor protein and key transducer of Dectin-1 signaling, in fungi-mediated MDSC generation in patients with genetic *CARD9* deficiency and *Card9* knockout mice. These approaches demonstrated that CARD9 signaling was involved in fungal MDSC induction in the human and the murine system (Figures 3E and 3F).

C. albicans induces interleukin-1 beta (IL-1β) in vitro (van de Veerdonk et al., 2009) and in vivo (Hise et al., 2009), which is critical for antifungal immunity (Vonk et al., 2006). Recent studies further provided evidence that IL-1β is involved in MDSC homeostasis (Bruchard et al., 2013). We observed an accumulation of intracellular IL-1β protein in $CD33^{+}$ myeloid cells followed by IL-1β release upon Dectin-1 ligand- and fungal-driven MDSC induction (Figure 4A). IL-1β protein, in turn, was sufficient to drive MDSC generation to a comparable extent as *C. albicans* did (Figure 4B). Studies in $Il1r^{-/-}$ mice, characterized by an increased susceptibility to *C. albicans* infection, demonstrated that abrogation of IL-1R signaling decreased MDSC accumulation in vivo (Figures 4B and S4A), and IL-1R antagonism in patients with autoinflammatory diseases decreased MDSCs (Figure S4B). As the inflammasome is the major mechanism driving IL-1β generation in myeloid cells through caspase activities, we blocked caspases chemically. We observed that pan-caspase inhibition largely abolished fungi-induced MDSC generation, which was not recapitulated by caspase-1 inhibition (Figure 4C). We therefore focused on caspase-8, since Dectin-1 activation was shown to trigger IL-1β processing by a caspase-8-dependent mechanism (Ganesan et al., 2014; Gringhuis et al., 2012). Indeed, fungal MDSC induction was paralleled by a substantial increase of caspase-8 activity, and caspase-8 inhibition diminished fungal-induced IL-1β production (Figure 4C) and the potential of fungi to induce MDSCs (Figure 4C). Conversely, supplementing

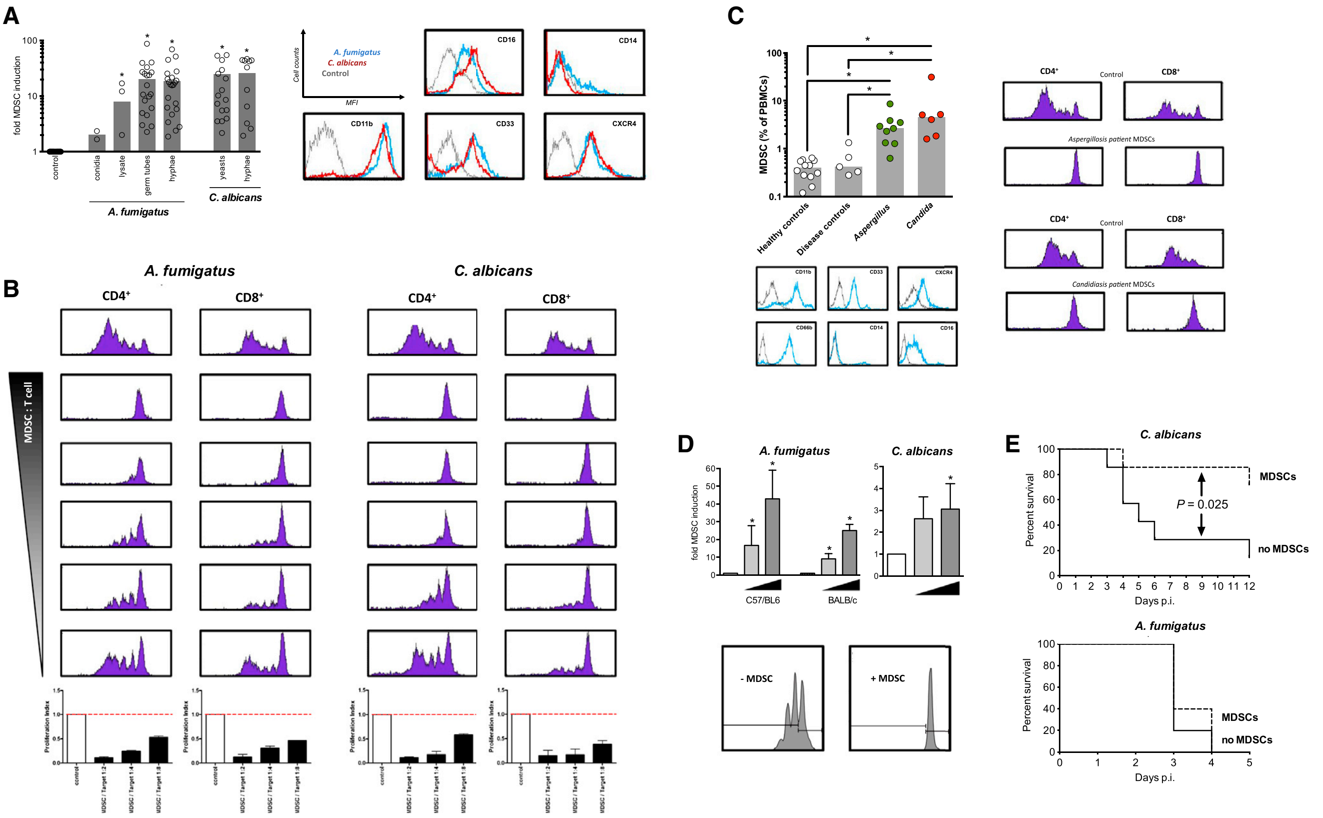

Figure 1. Fungi Induce Functional MDSCs In Vitro and In Vivo

(A) Fungal morphotypes differentially induce MDSCs.

Left panel: MDSCs were generated by incubating PBMCs (5×10^5/ml) from healthy donors with medium only (negative control), or different morphotypes of *A. fumigatus* (conidia, 5×10^5/ml; germ tubes, 1×10^5/ml; hyphae, 1×10^5/ml) or *C. albicans* (yeasts, 1×10^5/ml; hyphae, 1×10^5/ml). The x-fold induction of MDSCs compared to control conditions is depicted. $^*p < 0.05$.

Right panel: representative histograms of fungi-induced MDSCs ($CD11b^+CD33^+CD14^-CD16^+CXCR4^+$).

(B) Fungi-induced MDSCs suppress T cells. The suppressive effects of $CD33^+$-MACS-isolated MDSCs were analyzed on $CD4^+$ and $CD8^+$ T cell proliferation. MDSCs were generated by incubating PBMCs (5×10^5/ml) from healthy donors with *A. fumigatus* germ tubes (1×10^5/ml) or *C. albicans* yeasts (1×10^5/ml) for 6 days. Different MDSC-to-T cell ratios were assessed (1:2, 1:4, 1:6, 1:8, and 1:16). The lower bar graphs represent the proliferation index compared to control conditions as means ± SEM.

(C) MDSCs in patients with fungal infections.

Left panel: MDSCs were characterized as $CD14^-$ cells expressing CD33, CD66b, CD16, CD11b, and CXCR4 in the PBMC fraction. The gray line shows unstained controls. MDSCs were quantified in peripheral blood from healthy controls, immunosuppressed patients without fungal infections (disease controls, n = 5), or immunosuppressed patients with invasive fungal infections (invasive *A. fumigatus* infections, n = 9, and invasive *C. albicans* infections, n = 6). $^*p < 0.05$.

Right panel: representative CFSE stainings, showing the effect of MDSCs isolated (MACS) from patients with invasive *A. fumigatus* infections (left) or invasive *C. albicans* infections (right) on $CD4^+$ and $CD8^+$ T cell proliferation.

(D) Fungi induce MDSCs in mice in vivo.

Upper left panel: C57/BL6 (n = 3 mice per treatment group) or BALB/c (n = 4 mice per treatment group) wild-type mice were not infected (white bars) or challenged intranasally with 1×10^4 (light gray bar) or 1×10^6 (dark gray bar) *A. fumigatus* conidia for 3 days. On the fourth day, a bronchoalveolar lavage (BAL) was performed, and $CD11b^+Ly6G^+$ MDSCs were quantified by FACS. The x-fold induction of $CD11b^+Ly6G^+$ MDSCs in the BAL compared to control non-infected conditions is depicted. $^*p < 0.05$.

Upper right panel: C57BL/6 mice were not infected (white bars) or injected via the lateral tail vein with 2.5×10^5 (light gray bar) or 5×10^5 (dark gray bar) blastospores of *C. albicans*. On the fifth day, mice were sacrificed, and $CD11b^+Ly6G^+$ MDSCs in the spleen were quantified by FACS. The x-fold induction of $CD11b^+Ly6G^+$ MDSCs in the spleen compared to control non-infected conditions is depicted. n = 5 mice per treatment group. $^*p < 0.05$.

Lower panel: bone marrow-isolated murine $CD11b^+Ly6G^+$ MDSCs were co-cultured for 3 days with T cells ($CD4^+$ splenocytes) at a 1:2 (MDSCs:T cell) ratio. T cell proliferation was analyzed using the CFSE assay with and without MDSCs.

(E) Adoptive transfer of MDSCs modulates survival in fungal infection. For adoptive transfer experiments, $CD11b^+Ly6G^+$ MDSCs were isolated from the bone marrow of BALB/c mice by MACS and checked for T cell suppression. In (A)–(D) bars represent means ± SEM.

Upper panel: adoptive MDSC transfer was performed by intravenous (i.v.) injection of 5×10^6 MDSCs per animal. Seven mice received MDSCs, while seven mice served as non-MDSC control animals. A total of 2 hr after the MDSC transfer, mice were i.v. injected with 1×10^5 blastospores of *C. albicans*. Mice were weighed daily and monitored for survival and signs of morbidity.

Lower panel: for invasive pulmonary *A. fumigatus* infection survival studies, mice were immunosuppressed by treatment with cyclophosphamide, and MDSC transfer was performed by i.v. injection of 4×10^6 MDSCs per animal. Five mice received MDSCs, while five mice served as non-MDSC control animals. After the MDSC transfer, mice were challenged intranasally with 2×10^5 *A. fumigatus* conidia and were monitored for survival.

Cell Press

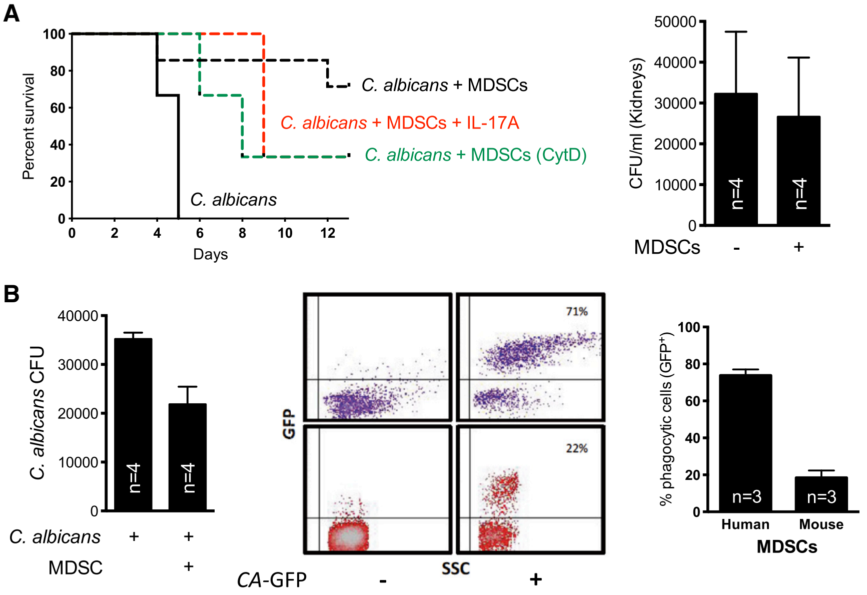

Figure 2. Antifungal Functions

(A) In vivo.

Left panel: survival in the invasive *C. albicans* infection model after adoptive MDSC transfer. Before adoptive transfer, isolated MDSCs were pretreated with cytochalasin D (CytD, 1 μg/ml, green line) or with recombinant mouse IL-17A protein (5 μg/mouse, red line).

Right panel: *C. albicans* CFUs in kidneys of BALB/c mice 5 days after adoptive transfer of MDSCs. Bars represent means ± SD.

(B) In vitro.

Left panel: 1×10^6 human MDSCs were co-cultured with 1×10^5 serum opsonized *C. albicans* (10:1 ratio) for 3 hr at 37°C in RPMI. Serial dilutions were performed of the cell suspension, and 100 μl was plated onto YPD agar plates containing penicillin and streptomycin. Plates were incubated for 24–48 hr at 37°C, and CFU were enumerated.

Middle and right panels: phagocytic capacity of human and murine MDSCs. Middle panel; upper (purple) FACS plots, isolated human granulocytic MDSCs (low-density $CD66b^+CD33^+$ cells) were co-cultured with or without GFP-labeled *C. albicans* (*CA*) spores (MOI = 1) in RPMI medium at 37°C for 90 min. Lower (red) FACS plots, isolated mouse granulocytic $CD11b^+Ly6G^+$ MDSCs were co-cultured with or without GFP-labeled *C. albicans* spores (MOI = 4) in RPMI medium at 37°C for 90 min. Representative dot blots are shown.

Right panel: GFP expression/fluorescence of MDSCs was analyzed by FACS and is given in the right panel as percentage of GFP^+ MDSCs.

IL-1β partially restored the abrogated MDSC generation upon caspase-8 inhibition (Figure S4C).

ROS are key factors in MDSC homeostasis (Gabrilovich and Nagaraj, 2009) and act downstream of Dectin-1 (Gross et al., 2009; Underhill et al., 2005). Therefore, we tested the involvement of ROS for fungal Dectin-1 ligand-induced MDSC generation using chemical inhibitors and cells from human CGD patients with ROS deficiency. These studies demonstrated that ROS contributed substantially to fungal MDSC induction (Figure 4D). Next, we investigated the interaction between ROS, caspase-8, and IL-1β and found that ROS inhibition dampened caspase-8 activity in response to fungi (Figure S4D). IL-1β, in turn, induced ROS production during MDSC culture, suggesting a positive feedback loop between caspase-8, IL-1β, and ROS in MDSC generation (Figures S4E and S4F).

DISCUSSION

While the complete genetic deletion of pro-inflammatory cytokines, particularly TNF-α, IL-1α/β, or IFN-γ, increases disease susceptibility in invasive fungal infections (Lionakis and Netea, 2013; Cheng et al., 2012; Gow et al., 2012; Netea et al., 2008, 2010), excessive inflammation causes collateral damage to the host (Carvalho et al., 2012; Romani et al., 2008), indicating that efficient protection against fungi requires a fine-tuned balance between pro-inflammatory effector and counter-regulatory immune mechanisms. Fungal infection induces an immunosuppressive state, and in murine models $CD80^+$ neutrophilic cells have been shown to be importantly involved in this process (Mencacci et al., 2002; Romani, 2011; Romani et al., 1997). By combining human and murine experimental systems, we extend this concept by providing evidence for an MDSC-mediated mechanism by which fungi modulate host defense, orchestrated by Dectin-1/CARD9, ROS, caspase-8, and IL-1β. This effect seems to be specific for neutrophilic MDSCs, since monocytic MDSCs were unchanged under our experimental conditions and were previously found to be downregulated by β-glucans in tumor-bearing mice (Tian et al., 2013).

C. albicans and *A. fumigatus* infections differ substantially with respect to T cell dependency and organ manifestation (Garcia-Vidal et al., 2013). Our finding that neutrophilic MDSCs were protective in a murine model of systemic *C. albicans* infection, but had no effect on pulmonary *A. fumigatus* infection, underlines this disparity and suggests MDSCs as a potential therapeutic approach in invasive *C. albicans*, rather than *A. fumigatus* infections. The MDSC-mediated effect was associated with downregulated NK and T cell activation, and Th17 responses and supplementing IL-17A in vivo could, at least partially, dampen the protective effect of MDSCs. Based on previous studies showing that NK cells drive hyperinflammation in candidiasis in immunocompetent mice (Quintin et al., 2014) and that IL-17 promotes fungal survival (Zelante et al., 2012), we speculate that MDSCs in fungal infections could act beneficial for the host by dampening pathogenic hyperinflammatory NK and Th17 responses (Romani et al., 2008; Zelante et al., 2007). Accordingly, enhancing neutrophilic MDSCs may represent an anti-inflammatory treatment strategy for fungal infections, particularly with *C. albicans*.

Recent studies put the gut in the center of immunotolerance. Dectin-1 was found to control colitis and intestinal Th17 responses through sensing of the fungal mycobiome (Iliev et al., 2012). The immunological events downstream of Dectin-1 and their functional impact on Th17 cells remained elusive. Our results demonstrate that fungal Dectin-1/CARD9 signaling induces MDSCs to dampen T cell responses and suggest that the immune homeostasis in the gut could be modulated by fungal-induced

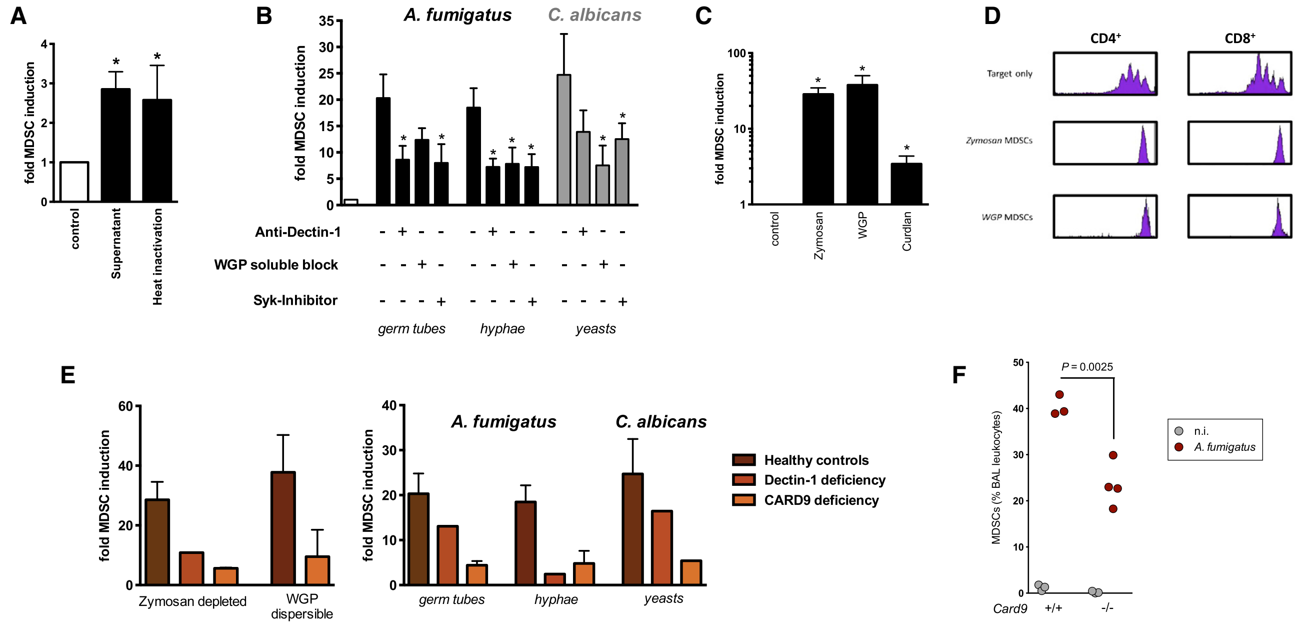

Figure 3. Fungi Induce MDSCs through a Dectin-1-, Syk-, and CARD9-Mediated Mechanism

(A) Fungal factors mediating MDSC induction are heat resistant. MDSCs were generated by incubating PBMCs (5×10^5/ml) from healthy donors with medium only (negative control), untreated, or heat-denatured (95°C, 30 min) supernatants (SNT) of *A. fumigatus* germ tubes (4%) for 6 days. The x-fold induction of MDSCs compared to control conditions is depicted. *$p < 0.05$ versus control conditions.

(B) Dectin-1 and Syk are involved in fungal MDSC induction. MDSCs were generated in vitro by incubating isolated PBMCs (5×10^5 cells/ml) with *A. fumigatus* germ tubes (1×10^5/ml), hyphae (1×10^5/ml), and *C. albicans* yeasts (1×10^5/ml) for 6 days. Where indicated, PBMCs were pretreated for 60 min with anti-Dectin-1 blocking antibody (15 μg/ml), soluble WGP (1 mg/ml), and a Syk inhibitor (100 nM). *$p < 0.05$ blocking versus unblocked conditions.

(C) Dectin-1/CARD9 ligands mimic fungal MDSC induction. MDSCs were generated in vitro by incubating isolated PBMCs with the Dectin-1/CARD9 ligands zymosan depleted (10 μg/ml), dispersible WGP (20 μg/ml), or curdlan (10 μg/ml). $p < 0.05$ versus control conditions.

(D) Dectin-1/CARD9 ligands induce functional MDSCs. The suppressive effects of $CD33^+$-MACS-isolated MDSCs were analyzed on $CD4^+$ and $CD8^+$ T cell proliferation (CFSE polyclonal proliferation assay). MDSCs were generated by incubating PBMCs (5×10^5/ml) from healthy donors with zymosan depleted (10 μg/ml) or dispersible WGP (20 μg/ml). MDSC, T cell ratio was 1:6.

(E) Fungal MDSC induction in patients with genetic Dectin-1 or CARD9 deficiency.

Left panel: MDSCs were generated in vitro by incubating isolated PBMCs (5×10^5 cells/ml) from healthy controls (n = 12), an individual with Dectin-1 deficiency, or patients with CARD9 deficiency (n = 2) with the Dectin-1/CARD9 ligands zymosan depleted (10 μg/ml) or dispersible WGP (20 μg/ml).

Right panel: MDSCs were generated in vitro by incubating isolated PBMCs (5×10^5 cells/ml) from healthy controls (n = 12), an individual with genetically proven Dectin-1 deficiency, or patients with CARD9 deficiency (n = 2) with different fungal morphotypes (1×10^5 cells/ml) for 6 days.

(F) CARD9 is involved in fungi-induced MDSC recruitment in vivo. *Card9*$^{-/-}$ mice and age-matched wild-type mice were challenged intranasally with 1×10^6 *A. fumigatus* conidia for 3 days. On the fourth day, a BAL was performed, and $CD11b^+Ly6G^+$ MDSCs were quantified by flow cytometry. In (B), (C), and (E) bars represent means ± SEM.

MDSCs. Beyond fungi, the Dectin-1/CARD9 pathway has been involved in bacterial and viral infections (Hsu et al., 2007), suggesting that this mechanism could play a broader role in balancing inflammation at host-pathogen interfaces.

EXPERIMENTAL PROCEDURES

Fungal Strains and Culture Conditions

A. fumigatus ATCC46645 conidia were incubated in RPMI at RT for 3 hr at 150 rpm to become swollen. Alternatively, conidia were cultured in RPMI overnight at RT, followed by germination in RPMI either at 37°C for 3 hr at 150 rpm to become germ tubes or at 37°C for 17 hr at 150 rpm to become hyphae. *C. albicans* SC5314 was grown on SAB agar plates at 25°C. One colony was inoculated and shaken at 200 rpm at 30°C in SAB broth overnight. To generate hyphae, live yeast forms of *C. albicans* were grown for 6 hr at 37°C in RPMI 1640. Killed yeasts and hyphae were prepared by heat treatment of the cell suspension at 95°C for 45 min or by fixing the cells for 1 hr with 4% paraformaldehyde followed by extensive washing with PBS to completely remove the fixing agent. The *C. albicans*-GFP *strain* TG6 was pre-cultured at 30°C, 200 rpm overnight in YPD medium.

Generation, Isolation, and Characterization of MDSCs

Neutrophilic MDSCs in peripheral blood were quantified based on their lower density and surface marker profiles as published previously (Rieber et al., 2013). Human MDSCs were generated in vitro according to a published protocol (Lechner et al., 2010). Murine MDSCs were characterized by CD11b, Ly6G, and Ly6C. Flow cytometry was performed on a FACS Calibur (BD Biosciences). Human and murine MDSCs were isolated using MACS (MDSC Isolation Kit; Miltenyi Biotec).

T Cell Suppression Assays

T cell suppression assays were performed as described previously (Rieber et al., 2013) using the CFSE method according to the manufacturer's protocol (Invitrogen).

Mouse Infection with *A. fumigatus* and *C. albicans*

Invasive *C. albicans* infection was established by IV injection in immunocompetent mice, whereas *A. fumigatus* infection was established by intranasal challenge in immunosuppressed mice. $CD11b^+Ly6G^+$ and $CD11b^+Ly6C^+$ cells in the spleens, BAL, and kidneys were quantified by FACS. For adoptive transfer experiments, $CD11b^+Ly6G^+$ MDSCs were isolated by MACS and transferred by IV injection of 4 or 5×10^6 MDSCs per animal.

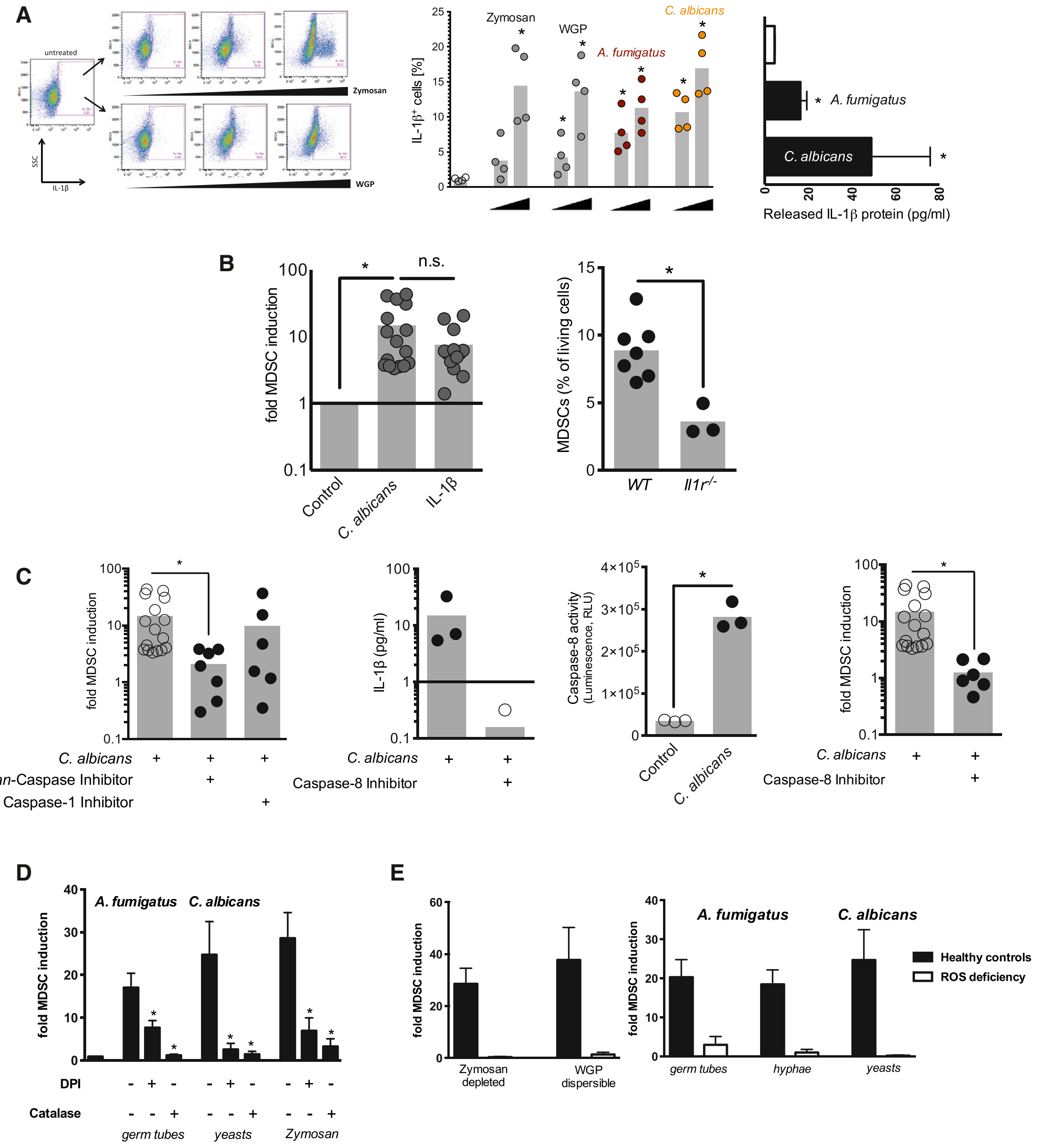

Figure 4. Fungal MDSC Induction Involves IL-1β, Caspase-8, and ROS

(A) Intracellular accumulation and release of IL-1β.

Left panel: gating strategy for intracellular cytokine staining. IL-1β was analyzed in $CD33^+$ myeloid cells using intracellular cytokine staining and flow cytometry. Zymosan depleted (20, 100, and 500 μg/ml) and WGP dispersible (20, 100, and 500 μg/ml) were used for 1 hr to stimulate cytokine production.

Middle panel: leukocytes isolated from healthy donors (n = 4) were left untreated (empty circles) or were treated for 1 hr with increasing concentrations of zymosan, WGP, *A. fumigatus* germ tubes, or *C. albicans* yeasts (each at 2×10^5/ml and 1×10^6/ml). IL-1β synthesis in $CD33^+$ cells was analyzed by intracellular cytokine stainings by flow cytometry. $^*p < 0.05$ versus control/untreated conditions.

Right panel: co-culture supernatants were collected after incubating isolated PBMCs (5×10^5 cells/ml) with medium only (white bar), *A. fumigatus* germ tubes (1×10^5 cells/ml), or *C. albicans* yeasts (1×10^5/ml) for 3 days. IL-1β was quantified by ELISA. $^*p < 0.05$ versus medium control conditions.

(B) IL-1β signaling is involved in fungal-induced MDSC generation.

Left panel: MDSCs were generated in vitro by incubating isolated PBMCs (5×10^5 cells/ml) with *C. albicans* yeasts (1×10^5/ml) or recombinant human IL-1β protein (0.01 μg/ml) for 6 days. $^*p < 0.05$.

Right panel: MDSCs ($CD11b^+Ly6G^+$) were quantified in spleens from *Il1r*$^{-/-}$ and age-matched WT mice 2 days after i.v. infection with 1×10^5 blastospores of *C. albicans*. $^*p < 0.05$.

(C) Fungal MDSC generation involves caspase-8. MDSCs were generated in vitro by incubating isolated PBMCs (5×10^5 cells/ml) with *C. albicans* yeasts (1×10^5/ml) for 6 days with or without pretreatment (where indicated) with the pan-caspase inhibitor Z-VAD-FMK (10 μM), the caspase-1 inhibitor Z-WEHD-FMK (50 μM), or the caspase-8 inhibitor Z-IETD-FMK (50 μM). IL-1β protein levels were quantified in cell culture supernatants by ELISA (note: two values were below detection limit). Caspase-8 activity was quantified in cell lysates using a luminescent assay. $^*p < 0.05$.

(legend continued on next page)

SUPPLEMENTAL INFORMATION

Supplemental Information includes four figures and Supplemental Experimental Procedures and can be found with this article online at http://dx.doi.org/10.1016/j.chom.2015.02.007.

AUTHOR CONTRIBUTIONS

N.R. and D.H. designed the study, supervised experiments, performed analyses, and wrote the manuscript. H.Ö., A.S., and M.C. performed murine infection studies. A.S., S.N.K., M.O., M. Ballbach, Y.Z., and I.S. performed MDSC in vitro assays. M. Bouzani and J. Loeffler performed and supervised NK cell assays. J. Loeffler and S.K. provided fungi, contributed to the design of the study, and wrote the manuscript. J.A. and A.B. performed and analyzed murine infection studies. R.H., M.M., J. Loeffler, J. Liese, A.N.R.W., M.E., R.S., H.R.S., C.S., L.M., and B.G. co-designed the study, provided patient material, and wrote the manuscript. J.R. and G.D.B. provided mice and co-designed in vivo experiments.

ACKNOWLEDGMENTS

We thank Gundula Notheis, University of Munich, and Thomas Lehrnbecher, University of Frankfurt, for patient samples. We thank Manfred Kneilling, University of Tübingen, for *Il1r*$^{-/-}$ mice. *Dectin-1*$^{-/-}$ mice were from Uwe Ritter, University of Regensburg, and originally generated by Gordon Brown, University of Aberdeen. We thank Steffen Rupp, Fraunhofer IGB Stuttgart, for the *C. albicans*-GFP strain TG6. This work was supported by the German Research Foundation (Deutsche Forschungsgemeinschaft, Emmy Noether Programme HA 5274/3-1 to D.H., the CRC/SFB685 to D.H. and A.N.R.W., and the TR/CRC124 FungiNet to A.B. and J. Loeffler), the Deutsche José Carreras Leukämie-Stiftung (DJCLS R 10/15 to A.B.), and the UK Wellcome Trust (to G.D.B.).

Received: September 30, 2014
Revised: December 17, 2014
Accepted: January 26, 2015
Published: March 12, 2015

REFERENCES

Aimanianda, V., Bayry, J., Bozza, S., Kniemeyer, O., Perruccio, K., Elluru, S.R., Clavaud, C., Paris, S., Brakhage, A.A., Kaveri, S.V., et al. (2009). Surface hydrophobin prevents immune recognition of airborne fungal spores. Nature *460*, 1117–1121.

Brown, G.D. (2010). How fungi have shaped our understanding of mammalian immunology. Cell Host Microbe *7*, 9–11.

Bruchard, M., Mignot, G., Derangère, V., Chalmin, F., Chevriaux, A., Végran, F., Boireau, W., Simon, B., Ryffel, B., Connat, J.L., et al. (2013). Chemotherapy-triggered cathepsin B release in myeloid-derived suppressor cells activates the Nlrp3 inflammasome and promotes tumor growth. Nat. Med. *19*, 57–64.

Carvalho, A., Cunha, C., Iannitti, R.G., De Luca, A., Giovannini, G., Bistoni, F., and Romani, L. (2012). Inflammation in aspergillosis: the good, the bad, and the therapeutic. Ann. N Y Acad. Sci. *1273*, 52–59.

Cheng, S.C., Joosten, L.A., Kullberg, B.J., and Netea, M.G. (2012). Interplay between *Candida albicans* and the mammalian innate host defense. Infect. Immun. *80*, 1304–1313.

Gabrilovich, D.I., and Nagaraj, S. (2009). Myeloid-derived suppressor cells as regulators of the immune system. Nat. Rev. Immunol. *9*, 162–174.

Ganesan, S., Rathinam, V.A., Bossaller, L., Army, K., Kaiser, W.J., Mocarski, E.S., Dillon, C.P., Green, D.R., Mayadas, T.N., Levitz, S.M., et al. (2014). Caspase-8 modulates dectin-1 and complement receptor 3-driven IL-1β production in response to β-glucans and the fungal pathogen, *Candida albicans*. J. Immunol. *193*, 2519–2530.

Garcia-Vidal, C., Viasus, D., and Carratalà, J. (2013). Pathogenesis of invasive fungal infections. Curr. Opin. Infect. Dis. *26*, 270–276.

Garlanda, C., Hirsch, E., Bozza, S., Salustri, A., De Acetis, M., Nota, R., Maccagno, A., Riva, F., Bottazzi, B., Peri, G., et al. (2002). Non-redundant role of the long pentraxin PTX3 in anti-fungal innate immune response. Nature *420*, 182–186.

Gow, N.A., van de Veerdonk, F.L., Brown, A.J., and Netea, M.G. (2012). *Candida albicans* morphogenesis and host defence: discriminating invasion from colonization. Nat. Rev. Microbiol. *10*, 112–122.

Gringhuis, S.I., Kaptein, T.M., Wevers, B.A., Theelen, B., van der Vlist, M., Boekhout, T., and Geijtenbeek, T.B. (2012). Dectin-1 is an extracellular pathogen sensor for the induction and processing of IL-1β via a noncanonical caspase-8 inflammasome. Nat. Immunol. *13*, 246–254.

Gross, O., Poeck, H., Bscheider, M., Dostert, C., Hannesschläger, N., Endres, S., Hartmann, G., Tardivel, A., Schweighoffer, E., Tybulewicz, V., et al. (2009). Syk kinase signalling couples to the Nlrp3 inflammasome for anti-fungal host defence. Nature *459*, 433–436.

Hise, A.G., Tomalka, J., Ganesan, S., Patel, K., Hall, B.A., Brown, G.D., and Fitzgerald, K.A. (2009). An essential role for the NLRP3 inflammasome in host defense against the human fungal pathogen *Candida albicans*. Cell Host Microbe *5*, 487–497.

Hohl, T.M., Van Epps, H.L., Rivera, A., Morgan, L.A., Chen, P.L., Feldmesser, M., and Pamer, E.G. (2005). *Aspergillus fumigatus* triggers inflammatory responses by stage-specific beta-glucan display. PLoS Pathog. *1*, e30.

Hsu, Y.M., Zhang, Y., You, Y., Wang, D., Li, H., Duramad, O., Qin, X.F., Dong, C., and Lin, X. (2007). The adaptor protein CARD9 is required for innate immune responses to intracellular pathogens. Nat. Immunol. *8*, 198–205.

Iliev, I.D., Funari, V.A., Taylor, K.D., Nguyen, Q., Reyes, C.N., Strom, S.P., Brown, J., Becker, C.A., Fleshner, P.R., Dubinsky, M., et al. (2012). Interactions between commensal fungi and the C-type lectin receptor Dectin-1 influence colitis. Science *336*, 1314–1317.

Kreindler, J.L., Steele, C., Nguyen, N., Chan, Y.R., Pilewski, J.M., Alcorn, J.F., Vyas, Y.M., Aujla, S.J., Finelli, P., Blanchard, M., et al. (2010). Vitamin D3 attenuates Th2 responses to *Aspergillus fumigatus* mounted by CD4+ T cells from cystic fibrosis patients with allergic bronchopulmonary aspergillosis. J. Clin. Invest. *120*, 3242–3254.

Lechner, M.G., Liebertz, D.J., and Epstein, A.L. (2010). Characterization of cytokine-induced myeloid-derived suppressor cells from normal human peripheral blood mononuclear cells. J. Immunol. *185*, 2273–2284.

Lionakis, M.S., and Netea, M.G. (2013). *Candida* and host determinants of susceptibility to invasive candidiasis. PLoS Pathog. *9*, e1003079.

Lionakis, M.S., Lim, J.K., Lee, C.C., and Murphy, P.M. (2011). Organ-specific innate immune responses in a mouse model of invasive candidiasis. J. Innate Immun. *3*, 180–199.

Lionakis, M.S., Swamydas, M., Fischer, B.G., Plantinga, T.S., Johnson, M.D., Jaeger, M., Green, N.M., Masedunskas, A., Weigert, R., Mikelis, C., et al.

(D) Fungal MDSC-inducing capacity is ROS dependent. MDSCs were generated in vitro by incubating isolated PBMCs (5×10^5 cells/ml) with different fungal morphotypes (1×10^5 cells/ml) or zymosan (10 μg/ml) for 6 days. PBMCs were pretreated where indicated with the NADPH oxidase inhibitor DPI (0.1 μM) or the H_2O_2 converting enzyme catalase (100 U/l). *$p < 0.05$ blocking versus unblocked conditions.

(E) Fungal MDSC induction in patients with ROS deficiency.

Left panel: MDSCs were generated in vitro by incubating isolated PBMCs (5×10^5 cells/ml) from healthy controls (n = 12) or patients with CGD (n = 3) with the Dectin-1/CARD9 ligands zymosan depleted (10 μg/ml) or dispersible WGP (20 μg/ml).

Right panel: MDSCs were generated in vitro by incubating isolated PBMCs (5×10^5 cells/ml) from healthy controls (n = 12) or CGD patients (n = 3) with different fungal morphotypes (1×10^5 cells/ml) for 6 days.

In (A)–(E) bars represent means ± SEM.

(2013). CX3CR1-dependent renal macrophage survival promotes *Candida* control and host survival. J. Clin. Invest. *123*, 5035–5051.

Mencacci, A., Montagnoli, C., Bacci, A., Cenci, E., Pitzurra, L., Spreca, A., Kopf, M., Sharpe, A.H., and Romani, L. (2002). CD80+Gr-1+ myeloid cells inhibit development of antifungal Th1 immunity in mice with candidiasis. J. Immunol. *169*, 3180–3190.

Moyes, D.L., Runglall, M., Murciano, C., Shen, C., Nayar, D., Thavaraj, S., Kohli, A., Islam, A., Mora-Montes, H., Challacombe, S.J., and Naglik, J.R. (2010). A biphasic innate immune MAPK response discriminates between the yeast and hyphal forms of *Candida albicans* in epithelial cells. Cell Host Microbe *8*, 225–235.

Netea, M.G., Brown, G.D., Kullberg, B.J., and Gow, N.A.R. (2008). An integrated model of the recognition of *Candida albicans* by the innate immune system. Nat. Rev. Microbiol. *6*, 67–78.

Netea, M.G., Simon, A., van de Veerdonk, F., Kullberg, B.J., Van der Meer, J.W., and Joosten, L.A. (2010). IL-1beta processing in host defense: beyond the inflammasomes. PLoS Pathog. *6*, e1000661.

Quintin, J., Voigt, J., van der Voort, R., Jacobsen, I.D., Verschueren, I., Hube, B., Giamarellos-Bourboulis, E.J., van der Meer, J.W., Joosten, L.A., Kurzai, O., and Netea, M.G. (2014). Differential role of NK cells against *Candida albicans* infection in immunocompetent or immunocompromised mice. Eur. J. Immunol. *44*, 2405–2414.

Rieber, N., Brand, A., Hector, A., Graepler-Mainka, U., Ost, M., Schäfer, I., Wecker, I., Neri, D., Wirth, A., Mays, L., et al. (2013). Flagellin induces myeloid-derived suppressor cells: implications for *Pseudomonas aeruginosa* infection in cystic fibrosis lung disease. J. Immunol. *190*, 1276–1284.

Romani, L. (2011). Immunity to fungal infections. Nat. Rev. Immunol. *11*, 275–288.

Romani, L., Mencacci, A., Cenci, E., Del Sero, G., Bistoni, F., and Puccetti, P. (1997). An immunoregulatory role for neutrophils in CD4+ T helper subset selection in mice with candidiasis. J. Immunol. *158*, 2356–2362.

Romani, L., Fallarino, F., De Luca, A., Montagnoli, C., D'Angelo, C., Zelante, T., Vacca, C., Bistoni, F., Fioretti, M.C., Grohmann, U., et al. (2008). Defective tryptophan catabolism underlies inflammation in mouse chronic granulomatous disease. Nature *451*, 211–215.

Romani, L., Zelante, T., De Luca, A., Bozza, S., Bonifazi, P., Moretti, S., D'Angelo, C., Giovannini, G., Bistoni, F., Fallarino, F., et al. (2009). Indoleamine 2,3-dioxygenase (IDO) in inflammation and allergy to *Aspergillus*. Med. Mycol. *47* (*Suppl 1*), S154–S161.

Spellberg, B., Johnston, D., Phan, Q.T., Edwards, J.E., Jr., French, S.W., Ibrahim, A.S., and Filler, S.G. (2003). Parenchymal organ, and not splenic, immunity correlates with host survival during disseminated candidiasis. Infect. Immun. *71*, 5756–5764.

Spellberg, B., Ibrahim, A.S., Edwards, J.E., Jr., and Filler, S.G. (2005). Mice with disseminated candidiasis die of progressive sepsis. J. Infect. Dis. *192*, 336–343.

Steele, C., Rapaka, R.R., Metz, A., Pop, S.M., Williams, D.L., Gordon, S., Kolls, J.K., and Brown, G.D. (2005). The beta-glucan receptor dectin-1 recognizes specific morphologies of *Aspergillus fumigatus*. PLoS Pathog. *1*, e42.

Tian, J., Ma, J., Ma, K., Guo, H., Baidoo, S.E., Zhang, Y., Yan, J., Lu, L., Xu, H., and Wang, S. (2013). β-glucan enhances antitumor immune responses by regulating differentiation and function of monocytic myeloid-derived suppressor cells. Eur. J. Immunol. *43*, 1220–1230.

Underhill, D.M., Rossnagle, E., Lowell, C.A., and Simmons, R.M. (2005). Dectin-1 activates Syk tyrosine kinase in a dynamic subset of macrophages for reactive oxygen production. Blood *106*, 2543–2550.

van de Veerdonk, F.L., Joosten, L.A., Devesa, I., Mora-Montes, H.M., Kanneganti, T.D., Dinarello, C.A., van der Meer, J.W., Gow, N.A., Kullberg, B.J., and Netea, M.G. (2009). Bypassing pathogen-induced inflammasome activation for the regulation of interleukin-1beta production by the fungal pathogen *Candida albicans*. J. Infect. Dis. *199*, 1087–1096.

Vonk, A.G., Netea, M.G., van Krieken, J.H., Iwakura, Y., van der Meer, J.W., and Kullberg, B.J. (2006). Endogenous interleukin (IL)-1 alpha and IL-1 beta are crucial for host defense against disseminated candidiasis. J. Infect. Dis. *193*, 1419–1426.

Werner, J.L., Metz, A.E., Horn, D., Schoeb, T.R., Hewitt, M.M., Schwiebert, L.M., Faro-Trindade, I., Brown, G.D., and Steele, C. (2009). Requisite role for the dectin-1 beta-glucan receptor in pulmonary defense against *Aspergillus fumigatus*. J. Immunol. *182*, 4938–4946.

Zelante, T., De Luca, A., Bonifazi, P., Montagnoli, C., Bozza, S., Moretti, S., Belladonna, M.L., Vacca, C., Conte, C., Mosci, P., et al. (2007). IL-23 and the Th17 pathway promote inflammation and impair antifungal immune resistance. Eur. J. Immunol. *37*, 2695–2706.

Zelante, T., Iannitti, R.G., De Luca, A., Arroyo, J., Blanco, N., Servillo, G., Sanglard, D., Reichard, U., Palmer, G.E., Latgè, J.P., et al. (2012). Sensing of mammalian IL-17A regulates fungal adaptation and virulence. Nat. Commun. *3*, 683.

Cell Host & Microbe, Volume *17*

Supplemental Information

Pathogenic Fungi Regulate Immunity by Inducing Neutrophilic Myeloid-Derived Suppressor Cells

Nikolaus Rieber, Anurag Singh, Hasan Öz, Melanie Carevic, Maria Bouzani, Jorge Amich, Michael Ost, Zhiyong Ye, Marlene Ballbach, Iris Schäfer, Markus Mezger, Sascha N. Klimosch, Alexander N.R. Weber, Rupert Handgretinger, Sven Krappmann, Johannes Liese, Maik Engeholm, Rebecca Schüle, Helmut Rainer Salih, Laszlo Marodi, Carsten Speckmann, Bodo Grimbacher, Jürgen Ruland, Gordon D. Brown, Andreas Beilhack, Juergen Loeffler, and Dominik Hartl

Supplemental Data
Figure S1, related to Figure 1

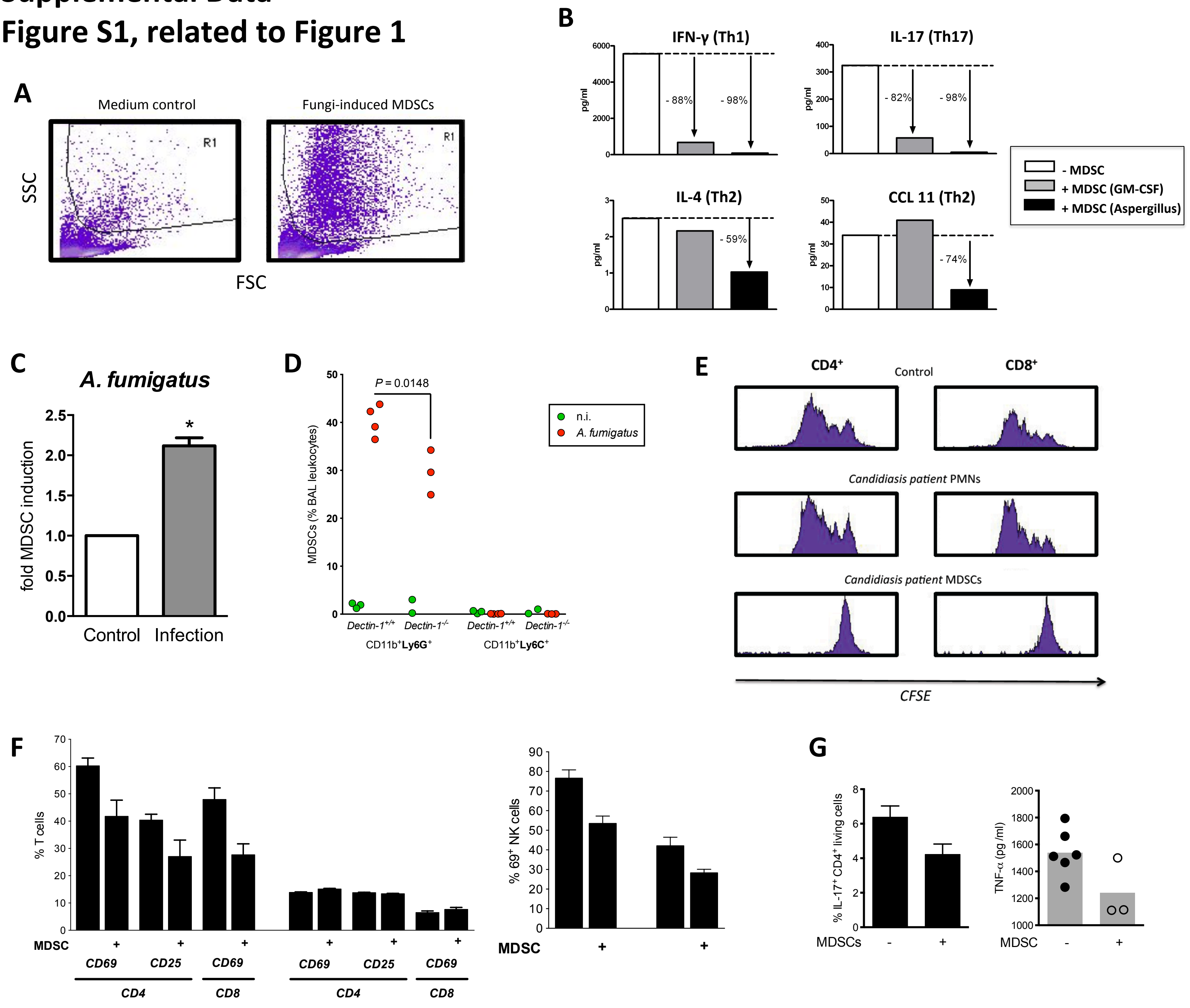

Figure S1. Phenotypic and functional MDSC characteristics

A. FSC/SSC characteristics of fungi-induced MDSCs *in vitro*
MDSCs were generated by incubating PBMCs (5 x 10^5 /ml) from healthy donors with medium only (negative control, ‘Medium control’) or *A. fumigatus* germ tubes (1 x 10^5 /ml, ‘Fungi-induced MDSCs’) for 6 days. Dot blots show representative MDSC gatings for subsequent immunophenotyping based on surface marker expression profiles as depicted in Figure 1a.

B. *Aspergillus*- and GM-CSF-induced MDSCs differentially affect Th1/Th17 and Th2 cytokine and chemokine levels
IL-2 and OKT-3 stimulated PBMCs were cultured in medium alone or together with Aspergillus- or GM-CSF- induced MDSCs for 96h. Cytokine and chemokine concentrations in culture supernatants were analyzed by multiplex array technology.

C. MDSC induction in immunodeficient mice
MDSC induction in immunodeficient mice: BALB/c wildtype mice were immunosuppressed with cyclophosphamide (150 mg/kg bw i.p.) and not infected (white bars) or challenged intranasally with 1x10^3 *A. fumigatus* conidia (grey bar) for three days. On the fourth day, $CD11b^+Ly6G^+$ MDSCs were quantified in lungs by FACS. The x-fold induction of $CD11b^+Ly6G^+$ MDSCs in the *A. fumigatus*–infected lung compared to control non-infected conditions is depicted. Bars represent means ± s.e.m. *$P<0.05$

D. MDSC induction in *Dectin-1*$^{-/-}$ mice
MDSC induction in *Dectin1*$^{-/-}$ mice: *Dectin-1*$^{-/-}$ mice and age-matched wildtype mice were challenged intranasally with 1x10^6 CFU *A. fumigatus* for three days. On the fourth day, a bronchoalveolar lavage was performed and granulocytic ($CD11b^+Ly6G^+$) or monocytic ($CD11b^+Ly6C^+$) MDSCs were quantified by flow cytometry.

E. *Candida*-induced neutrophilic MDSCs, but not conventional PMNs, suppress T-cell proliferation
Representative CFSE stainings, showing the effect of neutrophilic MDSCs or conventional autologous neutrophils (PMNs) isolated (MACS) from patients with invasive *C. albicans* infections on $CD4^+$ and $CD8^+$ T-cell proliferation.

F. Effect of MDSCs on inflammation
T- and NK-cell activation was quantified in kidneys (left bars) and spleens (right bars) (5 days p.i.) in the invasive *C. albicans* infection model with and without adoptive MDSC transfer. T and NK cell activation was measured by CD69 and CD25 surface expression on $CD4^+$ T cells, $CD8^+$ T cells and on $CD3^-DX5^+NKp46^+$ NK cells. T cell graph (left): the left bars show kidney, the right bars spleen. NK cell graph (right): the left bars show kidney, the right bars spleen.

G. IL-17A was stained intracellularly in $CD4^+$ splenocytes 5 days after adoptive MDSC transfer by flow cytometry. TNF-α protein levels were quantified in serum 5 days after adoptive MDSC transfer by Bioplex.

Figure S2,
related to Figure 1

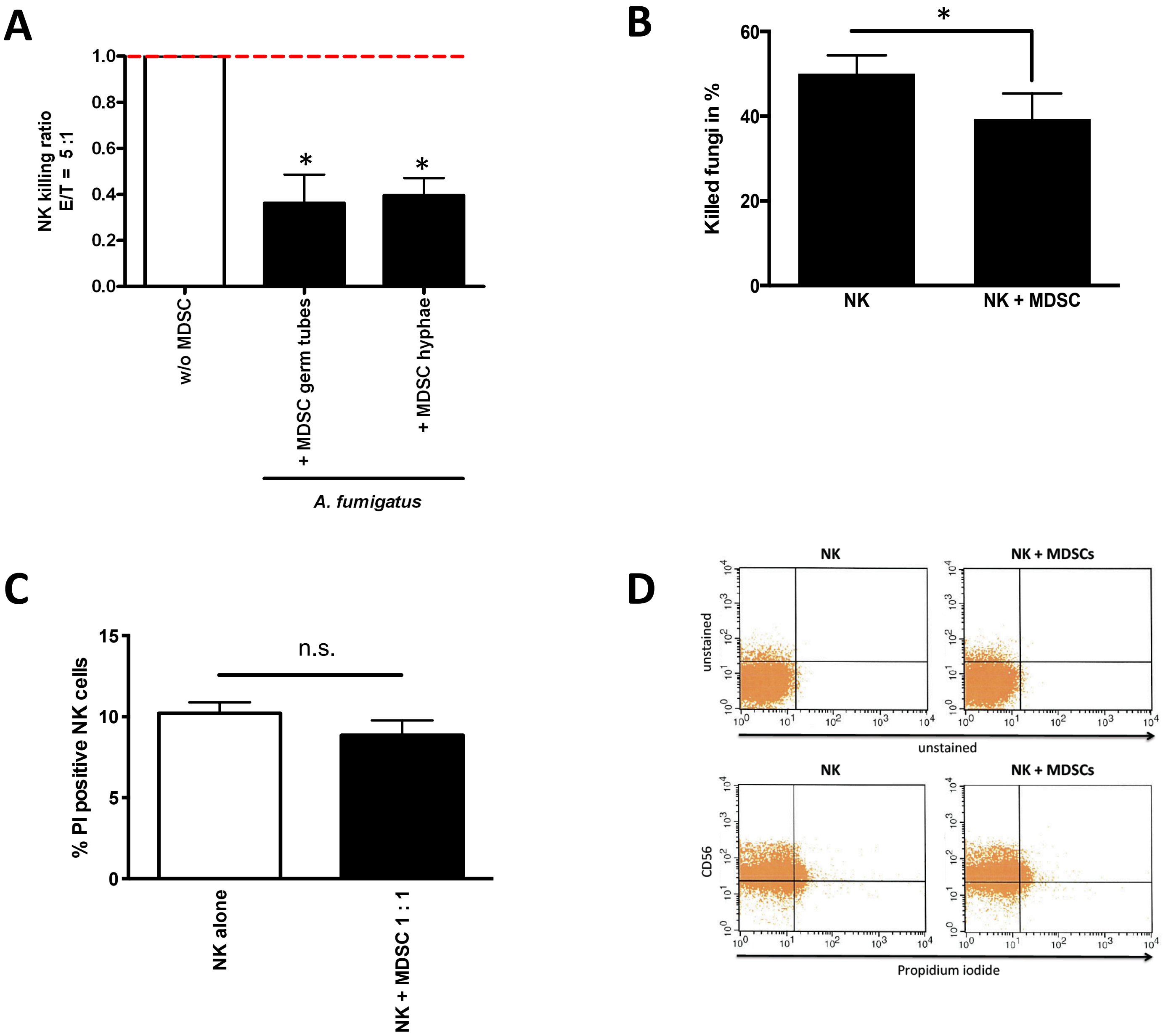

Figure S2: *Aspergillus*-induced MDSCs decrease antifungal NK killing activity

A. The suppressive effects of $CD33^+$-MACS-isolated MDSCs on NK cells were analyzed by measuring the NK cell cytotoxicity against K562 tumor cell line (europium release assay). MDSCs were generated by incubating PBMCs ($5x10^5$/ml) from healthy donors with *A. fumigatus* germ tubes ($1x10^5$/ml). MDSC to NK cell ratio was 1:1. NK (Effector, E) to K562 (Target, T) ratio was 5:1. Bars represent means ± s.e.m. **P*<0.05;

B. Activated NK cells were co-cultured with purified MDSC at a 1:1 ratio for 16h. After co-culture, MDSC were depleted and purified NK cells were incubated with *A. fumigatus* germ tubes at a 1:1 ratio for 5h. Fungal cell viability was determined using an XTT assay. **P*<0.05

C. MDSCs and NK cells were isolated from healthy PBMCs by magnetic bead technique. NK cells were cultured in medium alone or co-cultured together with MDSCs in a ratio of 1:1 overnight analogous to the cytotoxicity assays. Dead cells were stained with propidium iodide (PI). Bar graphs show percentages of PI positive dead NK cells within all NK cells. n.s. not significant

D. NK cell viability as assessed by propidium iodide staining for NK cells ($CD3^-CD56^+$ cells) in 1:1 NK-MDSC co-culture assays. The upper panel shows unstained controls from the same cells.

Figure S3, related to Figure 3

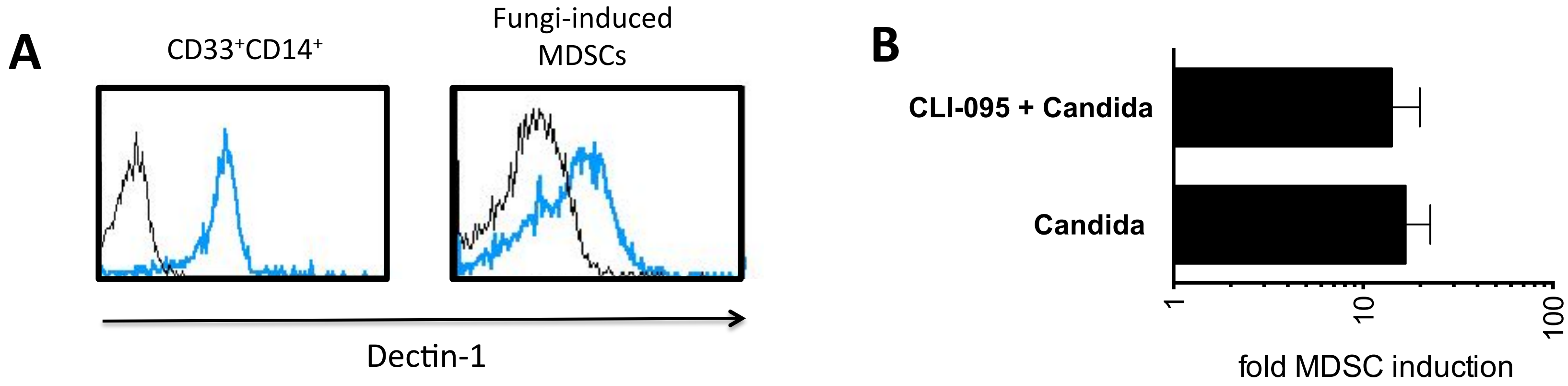

Figure S3: Dectin-1 and TLR4

A. Histograms show representative examples of Dectin-1 surface expression on fungi-induced MDSCs and $CD33^+CD14^+$ cells.

B. MDSCs were generated by incubating PBMCs ($5x10^5$/ml) from healthy donors with *C. albicans* (yeasts: $1x10^5$/ml) with or without 1h pretreatment with the TLR4 inhibitor CLI-095 (1µM). The x-fold induction of MDSCs compared to control conditions is depicted. Bars represent means ± s.e.m.

Figure S4, related to Figure 4

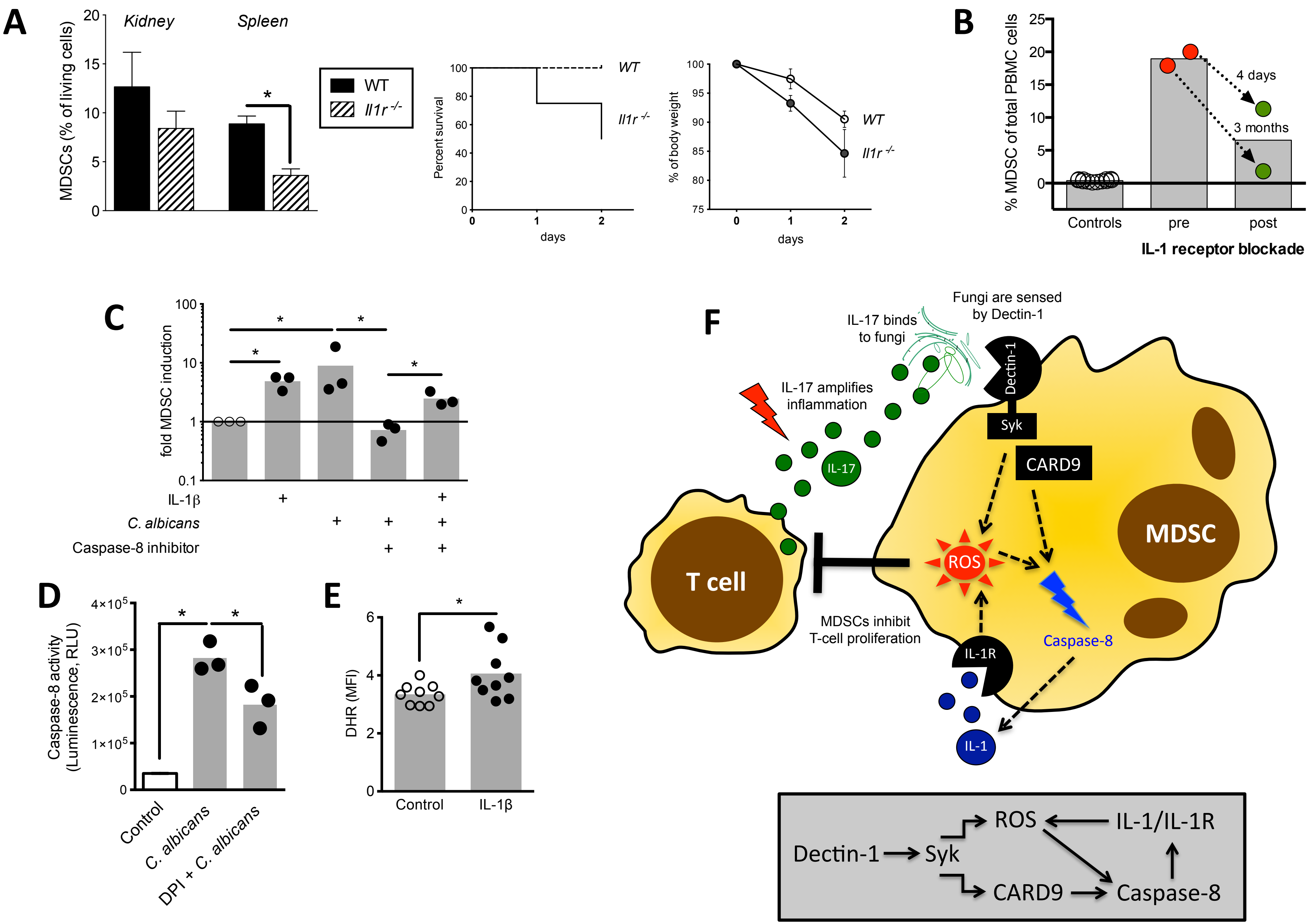

Figure S4: IL-1, Caspase-8 and ROS

A. WT and *Il1r*$^{-/-}$ mice were i.v. injected with 1×10^{5} blastospores of *Candida albicans* SC5314 per animal in 100 µl PBS. Mice were weighed daily and monitored for survival and weight loss. Mice with a weight loss of more than 20% or with serious symptoms of illness were euthanized. For MDSC quantification, mice were sacrificed and CD11b^{+}Ly6G^{+} MDSCs in the kidneys and spleens were quantified by FACS. Bars represent means ± s.e.m. *$P<0.05$

B. MDSCs were quantified in peripheral blood of two patients before and after systemic anti-IL-1 therapy using the IL1-receptor-antagonist *anakinra* (3 mg/kg bw/d). Patient 1 (male, 2 years of age, 3 months on *anakinra*) had a severe chronic non-classified autoinflammatory disease and patient 2 (female, 9 years of age, 4 days on *anakinra*) suffered from systemic onset juvenile idiopathic arthritis (soJIA).

C. MDSCs were generated *in vitro* by incubating isolated PBMCs (5x10^{5} cells/ml) with *C. albicans* yeasts (1x10^{5}/ml) or recombinant human IL-1β (0.01 µg/ml) for 6 days with or without pretreatment with the caspase-8 inhibitor Z-IETD-FMK (50µM). MDSCs were quantified using flow cytometry. Bars represent means. *$P<0.05$

D. MDSCs were generated *in vitro* by incubating isolated PBMCs (5x10^{5} cells/ml) with *C. albicans* yeasts (1x10^{5}/ml) for 6 days with or without pretreatment with the NADPH oxidase inhibitor DPI (0.1 µM). Caspase-8 activity was measured by a luminescent assay (Caspase-Glo 8 Assay from Promega, USA). Bars represent means. *$P<0.05$

E. PBMCs of healthy donors were treated with medium only (control) or recombinant human IL-1β (0.1 µg/ml) for 4 hours. After stimulation with PMA (200nM) for another 8 minutes, ROS production was measured by DHR in CD33^{+} myeloid cells. Bars represent means. *$P<0.05$

F. Proposed model of MDSC generation in invasive fungal infections: Fungal sensing through Dectin-1 triggers downstream signaling cascades involving Syk and CARD9, leading to caspase-8 activation. Caspase-8 drives interleukin-1 (IL-1) production. Released IL-1 binds to the IL-1 receptor (IL-1R) and enhances generation of ROS, which are essential for MDSC induction. Moreover, ROS are involved in fungal-driven caspase-8 activation. Generated MDSCs inhibit NK and T-cell responses, such as Th17 responses that amplify inflammation and may also directly affect fungal survival.

Supplemental Experimental Procedures

Study subjects

The study was conducted at the University Children's Hospital Tübingen (Germany). Informed consent was obtained from all subjects included in the study and all study methods were approved by the local ethics committee. At time of blood sampling all healthy subjects were without signs of infection, inflammation, or respiratory symptoms. Nine patients with invasive aspergillosis (positive *Aspergillus* galactomannan serum test and clinical signs of invasive aspergillosis) and six patients with invasive *Candida* bloodstream infections were also included in the study after written informed consent. These patients acquired invasive fungal infection during oncologic chemotherapy, after hematopoietic stem cell transplantation or showed fungal endocarditis. Moreover, five immunosuppressed patients after hematopoietic stem cell transplantation, but without fungal infections, were included as disease control group. In two patients with autoinflammatory diseases, MDSCs were quantified in peripheral blood before and after treatment with the IL-1R antagonist anakinra (3mg/kg/d). We further obtained blood from two patients with CARD9 deficiency and a medical history of several invasive fungal infections. The CARD9 mutations were: c.883G>A(hom) and c.883C>T(hom), both resulting in a premature termination codon (Q295X) consistent with a previously defined CARD9 defect (Glocker et al., 2009). On protein level no CARD9 protein could be detected in the patient's neutrophils and monocytes. We analyzed blood from a healthy subject with a homozygous Dectin-1 stop codon mutation (Tyr238X) consistent with a previously described Dectin-1 deficiency (Ferwerda et al., 2009). This mutation was identified through a whole exome sequencing approach for his affected daughter. In addition, we obtained fresh blood samples from three patients with chronic granulomatous disease (CGD) and complete ROS deficiency.

A. fumigatus strain and culture conditions

A. fumigatus ATCC46645 conidia were frozen at -80°C in glycerol stocks. After growing on Sab agar plates at 37°C, one colony was inoculated into Sab broth and shaken at 37°C overnight. Conidia were incubated in RPMI at room temperature (RT) for 3 h at 150 rpm to become swollen. Alternatively, conidia were cultured in RPMI overnight at RT, followed by germination in RPMI either at 37°C for 3 h at 150 rpm to become germ tubes or at 37°C for 17h at 150 rpm to become hyphae. Fungi were washed twice in PBS and heat-inactivated for 30 min at 95°C. Culture supernatants from conidia, germ tubes and hyphae were centrifuged at 8000 rpm for 15 min, followed by steril filtration using a 0.2 µm filter, respectively.

C. albicans strain and culture conditions

C. albicans SC5314 was stored as frozen stocks in 35 % glycerol at –80 °C and routinely grown on Sabouraud (Sab) agar plates at 25°C. One colony was inoculated and shaken at 200 rpm at 30°C in Sab broth (1% mycological peptone and 4% glucose) overnight. Cells were harvested by centrifugation and washed twice in Dulbecco's phosphate-buffered saline (PBS). Cells were counted in a haemocytometer and density was adjusted to the desired concentration in either PBS or RPMI 1640 medium. To generate hyphae, live yeast forms of *C. albicans* were grown for 6 h at 37°C in RPMI 1640 (Gibco-BRL). Killed yeasts and hyphae were prepared by heat treatment of the cell suspension at 95°C for 45 minutes or by fixing the cells for 1 h with 4% paraformaldehyde followed by extensive washing with PBS to completely remove the fixing agent. The *C. albicans*-GFP *strain* TG6 (a generous gift from Dr. Steffen Rupp, Fraunhofer IGB Stuttgart) was pre-cultured at 30°C, 200 rpm overnight in YPD medium. Cells were washed twice with sterile PBS and counted using a haemocytometer prior to use.

In vitro generation and isolation of human MDSCs

Human MDSCs were generated *in vitro* according to a previously published protocol (Lechner et al., 2010). Isolated human PBMCs were cultured in 12 well flat-bottom plates (Corning) or 25 cm² flasks (Greiner Bio-One) at 5×10^5 cells /ml in complete medium for 6 d, and GM-CSF (10 ng/ml, Genzyme), heat inactivated (95°C, 30min) *A. fumigatus* morphotypes (1:1 to 1:5 Aspergillus / PBMC ratio), *A. fumigatus* lysates (Miltenyi Biotec), *A. fumigatus* culture supernatants (4%), heat or formaldehyde inactivated *C. albicans* yeast and hyphae (1:5 to 1:20 Candida / PBMC ratio), curdlan (10 µg/ml, Invivogen), depleted zymosan (10 µg/ml, Invivogen) and WGP dispersible (20 µg/ml, Invivogen) were added as indicated in the respective figures. For blocking/inhibition experiments mouse anti-human Dectin-1 blocking antibody (15 µg/ml, AbD Serotec), WGP soluble (1 mg/ml, Invivogen), small molecule *syk*-inhibitor (100 nM, Calbiochem), the pan-caspase inhibitor Z-VAD-FMK (10µM, R&D Systems), the caspase-8 inhibitor Z-IETD-FMK (50µM, R&D Systems), the caspase-1 inhibitor Z-WEHD-FMK (50µM, R&D Systems), DPI (0.1µM, Sigma-Aldrich), Catalase (100 U/l, Sigma-Aldrich), the TLR4 inhibitor CLI-095 (1µM, Invivogen) and/or cytochalasin D (2µg/ml; Enzo Life Sciences) were added as indicated in the respective figures. PBMCs cultured in medium alone were run in parallel as a control for each experiment. Medium and supplements were refreshed after three days. After six days, all cells were collected from PBMC cultures. Adherent cells were removed using non-protease cell detachment solution Detachin (Genlantis). MDSCs were characterized as $CD33^+CD11b^+CD16^+CD14^-$ cells using recently established species-specific MDSC markers (Rieber et al., 2013a; Rieber et al., 2013b). For functional studies $CD33^+$ MDSCs were isolated from each culture using anti-CD33 magnetic microbeads and LS column separation (Miltenyi Biotech) with two sequential separation steps according to manufacturer's instructions.

Flow cytometry

Neutrophilic MDSCs in peripheral blood were quantified as published previously by our group (Rieber et al., 2013a). Antibodies against human CD3, CD4, CD8, CD14, CD16, CD66b, HLA-DR and CXCR4 were purchased from BD Pharmingen. Antibodies against CD11b and CD33 were purchased from MiltenyiBiotec. Antibodies against Dectin-1 were purchased from R&D Systems. Mouse IgG1-FITC, Mouse IgM-FITC, Mouse IgG1-PE and Mouse IgG1-APC (BD Pharmingen) were used as isotype controls. Antibodies against mouse CD11b, Ly6G and Ly6C were from BD Biosciences, anti-mouse CXCR4 was from Biolegend. Anti-mouse CD4 and IL-17A were from Miltenyi Biotech. CD3, CD8, CD25, CD69, NKp46, DX5 and the corresponding isotype controls were from Biolegend. T cells were characterized by CD3, CD4, CD8, CD25 and CD69 stainings. NK were characterized by $CD3^-$, NKp46, DX5 and CD69 stainings. Where indicated, T- and NK- cell activation in mice were analyzed in spleen and kidney tissues. Leukocyte enrichment/isolation from kidney tissues was performed as described previously (Lionakis et al., 2011). In brief, kidneys were aseptically removed, finely minced and digested with Liberase TL and DNase (Roche) for 30 min with intermittent shaking at 37°C. Digested tissue was passed through a 70-μm filter, washed with sterile PBS and remaining red cells were lysed with lysis buffer. Resulting suspensions were passed through a 40-μm filter and washed with PBS. Pellet was resuspended in 8 ml of 40% Percoll (GE Healthcare). Leukocyte enrichment was performed by overlaying Percoll-cell suspension on 3 ml of 70% Percoll solution, and centrifugation at 2,000 rpm without brakes for 30 min at RT. The interphase was collected carefully, washed in PBS and suspended in FACS buffer. Cells were counted using a haematocytometer. Flow cytometry was performed on a FACS Calibur (BD). Results were expressed as percent of positive cells and mean fluorescence intensity (MFI). Calculations were performed with BD CellQuestPro analysis software.

T-cell suppression assays

T-cell suppression assays were performed as described previously by us in detail (Rieber et al., 2013a). Responder-PBMCs were obtained from healthy volunteers and stained with carboxyfluoresceinsuccinimidyl ester (CFSE) according to the manufacturer's protocol (Invitrogen). PBMCs were stimulated with 100 U/ml Interleukin-2 (IL-2; R&D Systems) and 1 µg/ml OKT3 (Janssen Cilag). In a standardized way, 60,000 PBMCs per well in RPMI1640 (Biochrom) were seeded in a 96-well microtitre plate and RPMI1640 only or 3,750 (1:16) to 30,000 (1:2) MDSCs in RPMI1640 were added. The cell culture was supplemented with 10% heat-inactivated human serum, 2mM glutamine, 100 IU/ml penicillin, and 100 mg/ml streptomycin. After 96h of incubation in a humidified atmosphere at 37°C and 5% CO_2 cells were harvested and supernatants were frozen in -20°C. For mouse T-cell suppression assays, $CD11b^+Ly6G^+$ MDSCs were isolated from bone-marrows using MACS (MDSC isolation kit, Miltenyi Biotec, Germany) and were co-cultured for three days (37°C, 5% CO_2) with T cells ($CD4^+$ splenocytes) at a 1:2 (MDSCs : T-cell) ratio. T cells were activated with CD3/CD28-beads (mouse T cell activation kit, Miltenyi Biotec, Germany) and recombinant mouse IL-2 (50 U/ml, Biolegend). The cell culture was supplemented with 10% fetal bovine serum and 2mM glutamine. CFSE-fluorescence intensity for human and murine assays was analyzed by flow cytometry to determine polyclonal T- cell proliferation.

Intracellular cytokine analysis

Erythrocytes were lysed with Pharm Lyse Buffer (BD Pharmingen), leukocytes were washed with cold PBS and resuspended in RPMI (3 ml) with supplements (10% human serum, 100 U/ml Penicillin, 100 U/ml Streptomycin, 2 mM L-glutamine; Gibco) with the addition of benzonase (50 U/ml; Promega). The cells were plated into a 96-well flat bottom plate (200 µl), stimulated as indicated and were cultured for one hour (37°C; 5% CO_2). Brefeldin A (Sigma) was added (c_{final} = 10 µg/ml) and cells were cultured overnight. The cells were harvested and washed with cold PBS (0.1% sodium azide). LIVE⁄DEAD Fixable Aqua was used to stain dead cells (Life Technologies). Fc-receptors were blocked with Flebogamma (50 µg/ml, Grifols Biologicals) and cells were stained extracellularly with anti-CD33 PerCP-Cy5.5 (BD Pharmingen). The cells were fixed and permeabilized with Cytofix/ Cytoperm (BD Pharmingen), Fc-receptors were blocked as before and IL-1β was stained intracellularly (eBioscience). Flow cytometry was performed on a FACS Canto II (BD Pharmingen). Results were expressed as percent of $CD33^+IL\text{-}1\beta^+$ cells. In murine infections models, IL-17A was stained intracellularly in $CD4^+$ splenocytes by flow cytometry as described previously by us (Mays et al., 2013). Calculations were performed with FlowJo analysis software (Tree Star).

Cytokine and Caspase analyses

IL-1β ELISA Kits (R&D systems) were used to quantify cytokine protein levels. Multiplex cytokine array analyses in human MDSC / PBMC co-culture supernatants and mouse serum were performed using human and mouse Bioplex protein multi-array systems (Bio-Rad). Caspase-8 activity in cell lysates was analysed using a luminescent assay (Caspase-Glo 8 Assay from Promega, USA). Assays were performed according to the manufacturer's recommendations.

NK cell cytotoxicity assay

NK cell cytotoxicity assays were performed as described previously by us (Rieber et al., 2013b). In brief, NK cells were separated by MACS and co-incubated with MDSCs for 16h in a 1:1 ratio. Afterwards cytolytic activity of NK cells against K562 tumor cell line was tested in a BATDA europium release. E:T ratio was 5:1. We used the ratios of NK cell cytotoxicity in the presence of MDSCs / NK cell cytotoxicity without MDSCs for statistical analysis. NK cell cytotoxicity without MDSCs was set to a fixed value of 1. For *A. fumigatus* killing, NK cells were pre-stimulated with 1000 IU IL-2 (MiltenyiBiotec) for 24h. Activated NK cells were co-cultured with purified MDSC at a 1:1 ratio for 16h. After co-culture, MDSC were depleted using a MACS separation column (MiltenyiBiotec) and purified NK cells were incubated with *A. fumigatus* germ tubes at a 1:1 ratio for 5h. NK cells were lysed using ddH2O and a cell viability assay (2,3-Bis-(2-methoxy-4-nitro-5-sulfophenyl)-2H-tetrazolium-5-carboxanilid [XTT] assay) was performed to determine killing of *A. fumigatus*. Propidium iodide was used to analyse NK cell death.

Fungal phagocytosis and killing assays

MDSCs were isolated by MACS separation and the phagocyte killing assay was performed as described previously for neutrophils (Bambach et al., 2009). Briefly, 1 × 10^6 MDSC were cocultured with 1 × 10^5 serum opsonized *C. albicans* (10:1 ratio) for 3 h at 37°C in RPMI. The cells were centrifuged, and suspended in sterile water for lysis. Serial dilutions were performed of the cell suspension and 100 µl was plated onto YPD agar plates containing penicillin and streptomycin. Plates were incubated for 24-48 h at 37°C and CFUs were enumerated. The phagocytic capacity of human and murine MDSCs was further assessed by FACS. Therefore, MACS-isolated human granulocytic MDSCs (low density $CD66b^+CD33^+$ cells) were co-cultured with GFP–labelled *C. albicans* spores (MOI=1) in RPMI medium at 37 °C for 90 min. MACS-isolated mouse granulocytic $CD11b^+Ly6G^+$ MDSCs were co-cultured with GFP–labelled *C. albicans* spores (MOI=4) in RPMI medium at 37°C for 90 minutes. GFP expression of MDSCs was analyzed by FACS.

Mouse infection with *A. fumigatus* and *C. albicans*

All animal studies were approved by the local authorities (TVA/RP IDs: AZ 35/9185.81-2 / K5/13). *A. fumigatus* conidia (strain ATCC46645) were harvested on the day of infection, submerged in 0.9% NaCl + 0.002% Tween-20, filtered, centrifuged for 10 min 3000 rpm and resuspended in 5 mL 0.9% NaCl + 0.002% Tween-20. *Card9*$^{-/-}$ mice on a C57/BL6 background, *Dectin-1*$^{-/-}$ mice on a BALB/c background or age-matched C57/BL6 or BALB/c WT mice, respectively, were challenged intranasally with $1x10^4$ or $1x10^6$ *A. fumigatus* conidia for three days. At the fourth day, a bronchoalveolar lavage (BAL) was performed and $CD11b^+Ly6G^+$ and $CD11b^+Ly6C^+$ cells were quantified in BAL fluid by FACS. *C. albicans* (strain SC5314) was grown at 30°C overnight in liquid YPD (yeast extract, peptone, and dextrose) medium containing penicillin and streptomycin. Cells were collected by centrifugation, washed and resuspended in PBS. Required cell density was adjusted using a haemocytometer. For infection, female C57BL/6 mice were injected via the lateral tail vein with $2.5×10^5$ or $5×10^5$ blastospores per animal in 200 µl PBS. Control animals were given PBS only. $CD11b^+Ly6G^+$ cells in the spleens were quantified by FACS. Where indicated, *C. albicans* infection experiments (see details above) were performed in *Il1r*$^{-/-}$ on a C57BL/6 background and matched C57BL/6 WT mice. For adoptive transfer experiments, $CD11b^+Ly6G^+$ MDSCs were isolated from the bone marrow of healthy female *BALB/c* mice by MACS (MDSC isolation kit, Miltenyi Biotec, Germany). Transfer was performed by injecting $4\text{-}5×10^6$ MDSCs per animal into eight to twelve weeks old (18–22 g) female *BALB/c* mice via lateral tail vein. Two hours after the MDSC transfer, mice were i.v. injected with $1×10^5$ blastospores of *C. albicans* (SC5314 in 100 µl PBS). Mice were weighed daily and monitored for survival and signs of morbidity. Mice with a weight loss of more than 20% were euthanized. For CFU determination, mice were euthanized at day 5 post-infection. The kidneys were aseptically removed, homogenized in 1ml PBS, serially diluted, and plated in duplicate on YPD agar containing penicillin and streptomycin. CFUs were determined after 48 hrs of incubation at 37°C. To assess the impact of phagocytosis *in vivo*, MDSCs were pretreated with Cytochalasin D (1µg/ml, Enzo Life Sciences) prior to adoptive transfer. Where indicated, recombinant mouse IL-17A protein (Biolegend) was mixed with *C. albicans* suspension and injected via mouse tail vein (5µg IL-17A protein/mouse). For invasive pulmonary *A. fumigatus* infection studies, eight to twelve weeks old (18–22 g) female *BALB/c* mice were immunosuppressed by treatment with cyclophosphamide (150 mg/kg bw i.p., days -3 and -1). Mice where challenged intranasally with $1x10^3$ or $2x10^5$ *A. fumigatus* conidia (freshly harvested from three days old plates). For survival studies, mice were challenged once with *A. fumigatus*, for MDSC induction studies for three consecutive days, as indicated in the respective figure legends. Where indicated, MDSC transfer was performed by intravenous injection of $4×10^6$ MDSCs per animal prior to infection and mice were monitored for survival as described above.

Statistical analysis

Statistical analysis was done using GraphPad Prism 5.0 (Graph Pad Software). Differences between the groups were determined by Students' t test. Survival was calculated using the Log-rank (Mantel-Cox) test. A *P* value of <0.05 was considered to be significant.

Supplemental References

Bambach, A., Fernandes, M.P., Ghosh, A., Kruppa, M., Alex, D., Li, D., Fonzi, W.A., Chauhan, N., Sun, N., Agrellos, O.A., *et al.* (2009). Goa1p of Candida albicans localizes to the mitochondria during stress and is required for mitochondrial function and virulence. Eukaryotic cell *8*, 1706-1720.

Ferwerda, B., Ferwerda, G., Plantinga, T.S., Willment, J.A., van Spriel, A.B., Venselaar, H., Elbers, C.C., Johnson, M.D., Cambi, A., Huysamen, C., *et al.* (2009). Human dectin-1 deficiency and mucocutaneous fungal infections. N Engl J Med *361*, 1760-1767.

Glocker, E.O., Hennigs, A., Nabavi, M., Schaffer, A.A., Woellner, C., Salzer, U., Pfeifer, D., Veelken, H., Warnatz, K., Tahami, F., *et al.* (2009). A Homozygous CARD9 Mutation in a Family with Susceptibility to Fungal Infections. New Engl J Med *361*, 1727-1735.

Lechner, M.G., Liebertz, D.J., and Epstein, A.L. (2010). Characterization of cytokine-induced myeloid-derived suppressor cells from normal human peripheral blood mononuclear cells. J Immunol *185*, 2273-2284.

Mays, L.E., Ammon-Treiber, S., Mothes, B., Alkhaled, M., Rottenberger, J., Muller-Hermelink, E.S., Grimm, M., Mezger, M., Beer-Hammer, S., von Stebut, E., *et al.* (2013). Modified Foxp3 mRNA protects against asthma through an IL-10-dependent mechanism. J Clin Invest *123*, 1216-1228.

Rieber, N., Brand, A., Hector, A., Graepler-Mainka, U., Ost, M., Schafer, I., Wecker, I., Neri, D., Wirth, A., Mays, L., *et al.* (2013a). Flagellin Induces Myeloid-Derived Suppressor Cells: Implications for Pseudomonas aeruginosa Infection in Cystic Fibrosis Lung Disease. J Immunol *190*, 1276-1284.

Rieber, N., Gille, C., Kostlin, N., Schafer, I., Spring, B., Ost, M., Spieles, H., Kugel, H.A., Pfeiffer, M., Heininger, V., *et al.* (2013b). Neutrophilic myeloid-derived suppressor cells in cord blood modulate innate and adaptive immune responses. Clin Exp Immunol *174*, 45-52.
